# Environmental Risk Score as a New Tool to Examine Multi-Pollutants in Epidemiologic Research: An Example from the NHANES Study Using Serum Lipid Levels

**DOI:** 10.1371/journal.pone.0098632

**Published:** 2014-06-05

**Authors:** Sung Kyun Park, Yebin Tao, John D. Meeker, Siobán D. Harlow, Bhramar Mukherjee

**Affiliations:** 1 Department of Epidemiology, University of Michigan School of Public Health, Ann Arbor, Michigan, United States of America; 2 Department of Environmental Health Sciences, University of Michigan School of Public Health, Ann Arbor, Michigan, United States of America; 3 Department of Biostatistics, University of Michigan School of Public Health, Ann Arbor, Michigan, United States of America; Stony Brook University, Graduate Program in Public Health, United States of America

## Abstract

**Objective:**

A growing body of evidence suggests that environmental pollutants, such as heavy metals, persistent organic pollutants and plasticizers play an important role in the development of chronic diseases. Most epidemiologic studies have examined environmental pollutants individually, but in real life, we are exposed to multi-pollutants and pollution mixtures, not single pollutants. Although multi-pollutant approaches have been recognized recently, challenges exist such as how to estimate the risk of adverse health responses from multi-pollutants. We propose an “Environmental Risk Score (ERS)” as a new simple tool to examine the risk of exposure to multi-pollutants in epidemiologic research.

**Methods and Results:**

We examined 134 environmental pollutants in relation to serum lipids (total cholesterol, high-density lipoprotein cholesterol (HDL), low-density lipoprotein cholesterol (LDL) and triglycerides) using data from the National Health and Nutrition Examination Survey between 1999 and 2006. Using a two-stage approach, stage-1 for discovery (n = 10818) and stage-2 for validation (n = 4615), we identified 13 associated pollutants for total cholesterol, 9 for HDL, 5 for LDL and 27 for triglycerides with adjustment for sociodemographic factors, body mass index and serum nutrient levels. Using the regression coefficients (weights) from joint analyses of the combined data and exposure concentrations, ERS were computed as a weighted sum of the pollutant levels. We computed ERS for multiple lipid outcomes examined individually (single-phenotype approach) or together (multi-phenotype approach). Although the contributions of ERS to overall risk predictions for lipid outcomes were modest, we found relatively stronger associations between ERS and lipid outcomes than with individual pollutants. The magnitudes of the observed associations for ERS were comparable to or stronger than those for socio-demographic factors or BMI.

**Conclusions:**

This study suggests ERS is a promising tool for characterizing disease risk from multi-pollutant exposures. This new approach supports the need for moving from a single-pollutant to a multi-pollutant framework.

## Introduction

Over the last several decades, numerous environmental pollutants have been examined as potential risk factors for various diseases and health responses. Most studies have focused on single pollutants, that is, examining a single factor or a set of species (e.g., arsenic species; polychlorinated biphenyl (PCB) congeners). However, in real life we are exposed to multiple pollutants and pollutant mixtures, not single pollutants. This complex exposure profile may have additive, synergistic or antagonistic effects which are not being detected by single pollutant approaches. In addition, the impact of combined exposures to multiple pollutants may differ from the sum of the impacts from single pollutant assessments [1].

A main issue of the single pollutant approach in epidemiologic research is that it is prone to confounding. For example, the health effects of PCBs are subject to confounding by methylmercury if participants were co-exposed to both toxicants from fish consumption. This example also suggests that beneficial nutrients such as omega-3 fatty acids may confound the toxic effects by PCBs and methylmercury [2], [3]. Therefore, a positive association in a single pollutant approach may be observed if the single pollutant is a proxy for other co-pollutants or a mixture of pollutants. Alternatively, if individual pollutants have relatively small effects but multiple pollutants as a whole influence the disease risk, the single-pollutant approach may not capture the true effects [4].

Recently, several studies have examined multiple pollutants. Patel and colleagues adopted an approach widely used in analyzing high-throughput genotype data, genome-wide association study (GWAS), and proposed an *Environment-Wide Association Study (EWAS)* to examined wide ranges of environmental factors including toxic chemicals as well as nutrients in relation to type-2 diabetes [5], lipid profiles [6], blood pressure [7] and all-cause mortality [8] using data from the National Health and Nutrition Examination Survey (NHANES). This systematic approach avoided a potential bias from selective reporting of subsets of analyses, outcomes, and adjustments [6]. Another EWAS approach which examined 76 environmental and lifestyle factors in relation to metabolic syndrome was conducted in Sweden [9]. Although these EWAS studies have yielded intriguing results, the statistical analyses were still based on single pollutant approaches. Multi-pollutant models were not considered. Of note, unlike GWAS with millions of markers, current EWAS studies have a moderate number of exposures and are not really comprehensive or “ultra high-dimensional” in nature. Similarly, misclassification, measurement error, temporal variations, and incomplete exposure data are inherent challenges to an EWAS study that modern genotyping techniques have overcome in GWAS.

Sun et al. [10] considered a number of statistical strategies to examine multiple pollutants and their interactions using regression methods for high-dimensional covariates, such as least absolute shrinkage and selection operator (LASSO) [11], Bayesian model averaging (BMA) [12] or supervised principal component analysis (SPCA) [13]. This study showed that LASSO and other dimension reduction techniques worked well for estimating risk models when a large number of candidate pollutants exist. Elastic-net method [14] or the adaptive elastic-net method [15] were proposed to take into account the issue of multi-collinearity when highly correlated predictors are fit simultaneously.

Another challenge in quantifying the health effects of multi-pollutant exposure is how to estimate the risk of adverse health responses from multiple pollutants. As stated above, single pollutant approaches and even EWAS in which the unit of analysis is based on a single pollutant have had small to modest effect sizes. The challenge is to construct the disease risk from exposure to multiple environmental risk factors [16]–[18]. Some advances have been made in the air pollution area (air pollution mixtures). For example, in the indicator approach one pollutant represents the combined exposure to several pollutants [19], [20]; or, in the source apportionment approach particle constituents are assigned to emission sources using principal component analysis and hierarchical clustering [21], [22]. However, these approaches do not account for a wide range of environmental pollutants.

In the general context of risk factor epidemiology, risk prediction models, such as the Framingham risk score for coronary heart disease [23] and genetic risk scores (a.k.a Genetic Risk Prediction Studies (GRIPS)) [24]–[29], have been widely used. Following from these ideas, it would be interesting to assess the predictive ability of an “*Environmental Risk Score*” as a follow-up to an EWAS study after identifying environmental pollutants significantly associated with health outcomes. A risk score may also facilitate targeting of preventive interventions [27].

Here, we propose an “Environmental Risk Score (ERS)” as a new tool to examine the risk of exposure to multi-pollutants in epidemiologic research. As a “proof of concept”, we used environmental biomonitoring data from NHANES to illustrate our methodology because it includes a wide range of environmental pollutants from representative U.S. populations and independent data from different cycles enabled us to discover and validate our findings. As outcomes, we examined serum lipid levels including total cholesterol, high-density lipoprotein cholesterol (HDL), low-density lipoprotein cholesterol (LDL) and triglycerides, because these are continuous measures that can be dichotomized at clinically relevant cutoff points, allowing us to evaluate both continuous and binary outcomes. These outcomes were used in the previous EWAS by Patel et al. [6]. We focused on environmental pollutants in this study rather than a broader array of environmental exposures including dietary, behavioral, psychosocial, socioeconomic and neighborhood, and microorganismic factors, which may limit the feasibility and applicability of ERS. Instead, we treated important determinants of lipid outcomes such as age, sex, race/ethnicity, education (an indicator of socioeconomic factor), body mass index (BMI), and selected dietary nutrients as covariates and confounding factors. The methodology can of course be generalized when the agnostic search for important predictors is expanded to a broader set of exposures capturing personal and community environment.

As the primary goal of the present study is to introduce this novel approach rather than to estimate and generalize actual risks in the U.S. population, and as some of the statistical procedures used in our approach are not equipped with automated handling of survey weights, we did not account for the complex sampling design and used conventional regression modeling. Biomonitoring data in NHANES were not measured in all participants; some pollutants were measured only in a subset (e.g., one third) and different kinds (classes) of pollutants were measured in different subsets in order to reduce the burden of examinations, which limits the sample size for this multi-pollutant model. To maximize the power of the proposed approach, we imputed unmeasured or missing pollutant data. For these reasons, our findings should be cautiously interpreted as potential associations. Another new feature of the present study is that we examined 4 lipid outcomes separately (single-phenotype approach) as well as all 4 lipid outcomes together as a whole (multi-phenotype approach). This multi-phenotype approach can also help improve the power to detect modest individual effects of environmental pollutants and reduce the burden of multiple testing [30]–[32].

## Methods

### Ethics Statement

NHANES is a publicly available data set and all participants in NHANES provide written informed consent, consistent with approval by the National Center for Health Statistics Institutional Review Board.

### Data

We obtained all publicly available data from the NHANES website (http://www.cdc.gov/nchs/nhanes.htm). Following the two-stage design as in genome-wide association studies [33], we selected three NHANES cycles, 1999–2000, 2001–2002, and 2005–2006 as stage 1 samples and NHANES 2003–2004 as stage 2 samples, because not all measures of environmental pollutants are available in all cycles and the 2003–2004 cycle had the largest number of shared pollutants. We restricted the sample to adults aged 20 years or older and did not include children in this study.

We focused on the 149 environmental pollutant variables that were measured in both stage 1 and 2 samples. The basic idea of an EWAS, like GWAS, is to conduct an agnostic search in a broad set of environmental compounds without any prior belief or hypothesis regarding the effects related to a given outcome. As our study was based on such a non-targeted approach and had no *a priori* assumption of the association directions, chemicals known to be less toxic, such as arsenosugars, were not screened out. For the concentrations below the National Centers for Health Statistics (NCHS) documented limit of detection (LOD), the values of each pollutant’s LOD/√2 were replaced. We eliminated 15 variables that had more than 90% of the observations missing (including missing due to below LOD), leaving 134 pollutants available for our analysis (Table S1). As stated above the four outcome variables included total cholesterol, HDL, LDL and triglycerides. Important covariates were chosen *a priori* and included age, sex, race/ethnicity (Mexican American, Other Hispanic, non-Hispanic white, non-Hispanic black, Other), education (categorized to less than high school diploma, high school diploma, and greater than high school diploma), BMI, and NHANES cycle. We selected education as an indicator of socioeconomic status because it is widely used and has less missing data than other proxies, such as household income or poverty income ratio. We also considered 21 blood measures of micronutrients (vitamins and isoflavone compounds), some of which were identified to predict serum lipids in the previous EWAS [6]. We imputed our data with a sequential imputation strategy using IVEWARE where the variables to be imputed were treated as the outcomes and all other variables were used as predictors [34], [35]. Since we used the data solely for an illustrative purpose, we used only one imputed dataset. The distributions of the data before and after imputation were similar (see File S1 for more details). The sample sizes after imputation were 10818 for the stage 1 sample and 4615 for the stage 2 sample. We applied logarithmic transformation with base 10 to the continuous outcomes and pollutant levels because of skewness in the distributions of the raw values.

### Discovery Process of Environmental Factors Contributing to ERS for Single Phenotype

#### 1. Choice of covariates and micronutrients

Our base model included age, gender, race/ethnicity, education and BMI as was also done by Patel et al. [5], [6]. Then we selected important micronutrients corresponding to each phenotype using the full data (stage 1 and 2 samples combined). Specifically, we first regressed each phenotype on the set of covariates in the base model to obtain the residuals, and then used the residuals as the outcome to select the micronutrients. For micronutrient selection we applied the Bayesian model averaging technique (BMA) to jointly analyze all micronutrients and select the ones with posterior inclusion probability greater than 0.8 (see Sun et al. [10] for details). Other simpler methods (e.g., best subset regression) may also be used at this step.

#### 2. Single-pollutant models

We selected environmental pollutants for each lipid outcome with adjustment for base covariates and outcome-specific micronutrients. Specifically, for subject *i* (*i* = 1, …, *N*), let *Y_i_* represent one given phenotype, *E_i_* be one given environmental pollutant, and *Z_i_* (*k*×1) be the vector of base covariates and micronutrients. The fitted single-pollutant model was
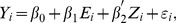
(1)where 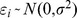. We adopted a two-stage analyses strategy following Skol et al. [36] using the model in (1). In stage 1, we analyzed the single-pollutant model for every pollutant using stage 1 samples and calculated the standard Wald test statistic *z*_1_ corresponding to 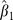. In stage 2, we only included pollutants with |*z*_1_| > *C*_1_ (pre-defined significance threshold). For each of these chosen pollutants, we repeated the same regression analysis using stage 2 samples, and calculated Wald test statistics *z*_2_ corresponding to 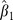. Finally, we conducted joint analysis to combine z_1_ and z_2_ and get a new statistic that allows for between-stage heterogeneity [36],
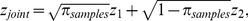
(2)where 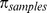 was the proportion of samples in stage 1 (0.7 in our case). *z_joint_* was compared with a significance threshold *C_joint_*. Thresholds *C_1_* and *C_joint_* were selected to control for the false positive rate. Details for the calculation can be found in Skol et al. [36]. Pollutants with |*z*_1_|>*C*_1_ and |*z_joint_*|>*C_joint_* were selected for ERS and in our study, we chose *C*_1_ and *C_joint_* to be 2.58 and 3.57, respectively (corresponding to a significance level of 0.01 for the Wald test in both stage 1 and stage 2 analyses). The choice of these thresholds can be optimized for enhanced power at a given false positive rate; however, we wanted to be liberal in the choice of these thresholds. Our primary goal was to identify pollutants to be included in the construction of the ERS that can be used for prediction of health risks, not just identification of individual pollutants, thus, we are less concerned about the false positive rate of the discovery process at this step. We denote the set of pollutants selected in this step as *E^s^*.

#### 3. Conditional analysis via multi-pollutant models

Motivated by the discovery strategy of additional genetic loci via conditioning on the loci identified through marginal association in GWAS [37], we further explored the possibility of identifying additional pollutants not selected in the previous two-stage analysis, in the presence of the previously selected ones in a multivariate model. Specifically, for subject *i*, let *E_i_^+^* denote a pollutant not belonging to *E^s^*. The conditional model is given by.
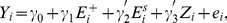
(3)where 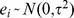. We repeated the two-stage analysis with this conditional model for each pollutant not belonging to *E^s^*. We calculated the same Wald test statistics and compared them to the same thresholds to select additional exposures for ERS. We denoted the set of pollutants selected in this step as *E^c^*, denoting pollutants identified based on conditional analysis.

### Construction of ERS and Assessment of its Predictive Power

We conceptualized the ERS as a weighted sum of the exposures identified by marginal and conditional analysis, namely, *E^s^* and *E^c^* i.e., for subject *i*, ERS*_i_*  =  *w^s’^E_i_^s^* + *w^c’^E_i_^c^*, where *w^s^* and *w^c^* are vectors of weights corresponding to *E^s^* and *E^c^*, respectively. Given that all exposure variables were log-transformed in the present study, the weights (regression coefficients) are on a relative (ratio) scale, not an absolute (difference) scale, and therefore the weights did not need to be scaled. For comparability of the weights on an absolute scale if exposure variables are linearly fit, they need to be scaled (by either standard deviation or IQR).

To estimate the weights and evaluate the performance of ERS, we randomly split the full data (all cycles combined) by a 3∶1 ratio: the larger part (n = 11586) used for estimation/training and the smaller part (n = 3847) for validation/testing. We considered two types of weights. ERS1 used regression coefficients from single-pollutant models for each pollutant in the *E^s^* and *E^c^* sets as weights, while ERS2 used regression coefficients from a multi-pollutant model that included all members of *E^s^* and *E^c^* simultaneously. The weights of ERS1 and ERS2 were both adjusted for base covariates and phenotype-specific micronutrients. ERS1 and ERS2 differ in terms of the weights corresponding to each pollutant, in particular, the weights in ERS2 are taking into account correlation among the pollutants in the entire *E^s^* and *E^c^* sets. We estimated the weights using the training data and calculated the ERS in the validation data based on those weights to avoid issues of over-fitting. We realize that the multiple regression model that includes both *E^s^* and *E^c^* with adjustment for base covariates and phenotype-specific micronutrients may have some redundant variables in terms of statistical significance, and a further variable selection step may lead to a smaller model and a more concise measure of ERS. We wanted to retain all the identified pollutants in both versions of ERS and thus refrained from applying this additional model selection step in constructing the weights from the multivariate model.

We evaluated the performance of ERS using three metrics. In each case, the contribution of ERS was measured in the presence of base covariates and micronutrients retained in the model. First, we used linear regression with the continuous phenotype outcome and continuous version of the ERS, with R^2^ and the predicted residual sums of squares (PRESS) statistic measuring model fit. Second, we dichotomized the levels of the phenotypes as high vs. low (200 mg/dL for total cholesterol; 40 mg/dL (male) or 50 mg/dL (female) for HDL; 130 mg/dL for LDL; and 150 mg/dL for triglycerides [38]), and conducted logistic regression analysis with this dichotomized outcome and with continuous ERS as predictor. We used area under the receiver operating characteristic (ROC) curve or AUC to assess predictive ability of the ERS with these binary endpoints. In each of the above two metrics we compared a sequence of models, with only base covariates, base covariates + micronutrients, base covariates + micronutrients + ERS. Note that the above two metrics measure overall prediction, aggregated over all subjects. A bootstrap resampling (2000 iterations) was used to compute 95% confidence intervals of AUCs for different models [39] (the ci.auc() function in the pROC package in R [40]).

In order to assess risk stratification/discrimination power of the ERS we further categorized ERS by its quintiles and conducted logistic regression for the binary phenotype and categorical ERS. We used the odds ratio (OR) for the highest quintile vs. the lowest quintile of ERS to measure the risk stratification properties of ERS.

### Extension to Multiple Phenotypes

Since we are dealing with multiple lipid outcomes that are correlated, a natural question may be to investigate whether simultaneously analyzing the phenotypes lead to methods with superior/different performance. In this step we used four phenotypes together to select environmental pollutants by multivariate regression. The micronutrients adjusted for were the union of all phenotype-specific micronutrients selected in Section 1. Specifically, for subject *i*, the multivariate single-pollutant model is.
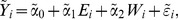
(4)where 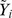 is the 4×1 vector of phenotypes, 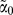 and 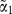
are 4×1 vectors of intercepts and regression coefficients for one given pollutant, respectively, 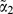 is the 4× *m* matrix of regression coefficients for base covariates and micronutrients *W*, (*m*×1) and 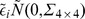. Similar to the single-phenotype method, we also applied the two-stage analysis. In stage 1, we analyzed the multivariate single-pollutant model for every pollutant using stage 1 samples and calculated the likelihood ratio Chi-squared test statistic with 4 degrees of freedom, namely, *χ_1_* comparing the multivariate single-pollutant model with the base model (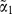 = 0). In stage 2, we repeated the same analysis using stage 2 samples, but only for pollutants with |*χ_1_*| > 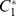 (pre-defined significance threshold), and calculated the same likelihood ratio test statistic *χ*_2_. We also used equation (2) (replace z with *χ*) to calculate *χ_joint_* which was compared with a significance threshold 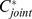. Again, thresholds 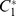 and 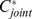 were selected to control the false positive rate and we set them to be 13.3 and 18.4, respectively (corresponding to a significance level of 0.01 for the chi-squared test with 4 degrees of freedom in each stage).

Similarly, we also conduct the conditional analysis using the multivariate multi-pollutant model adjusted for pollutants selected in the previous step, base covariates and micronutrients. We calculated the same likelihood ratio test statistics and compared them to the same thresholds to select additional exposures for ERS.

**Figure 1:**
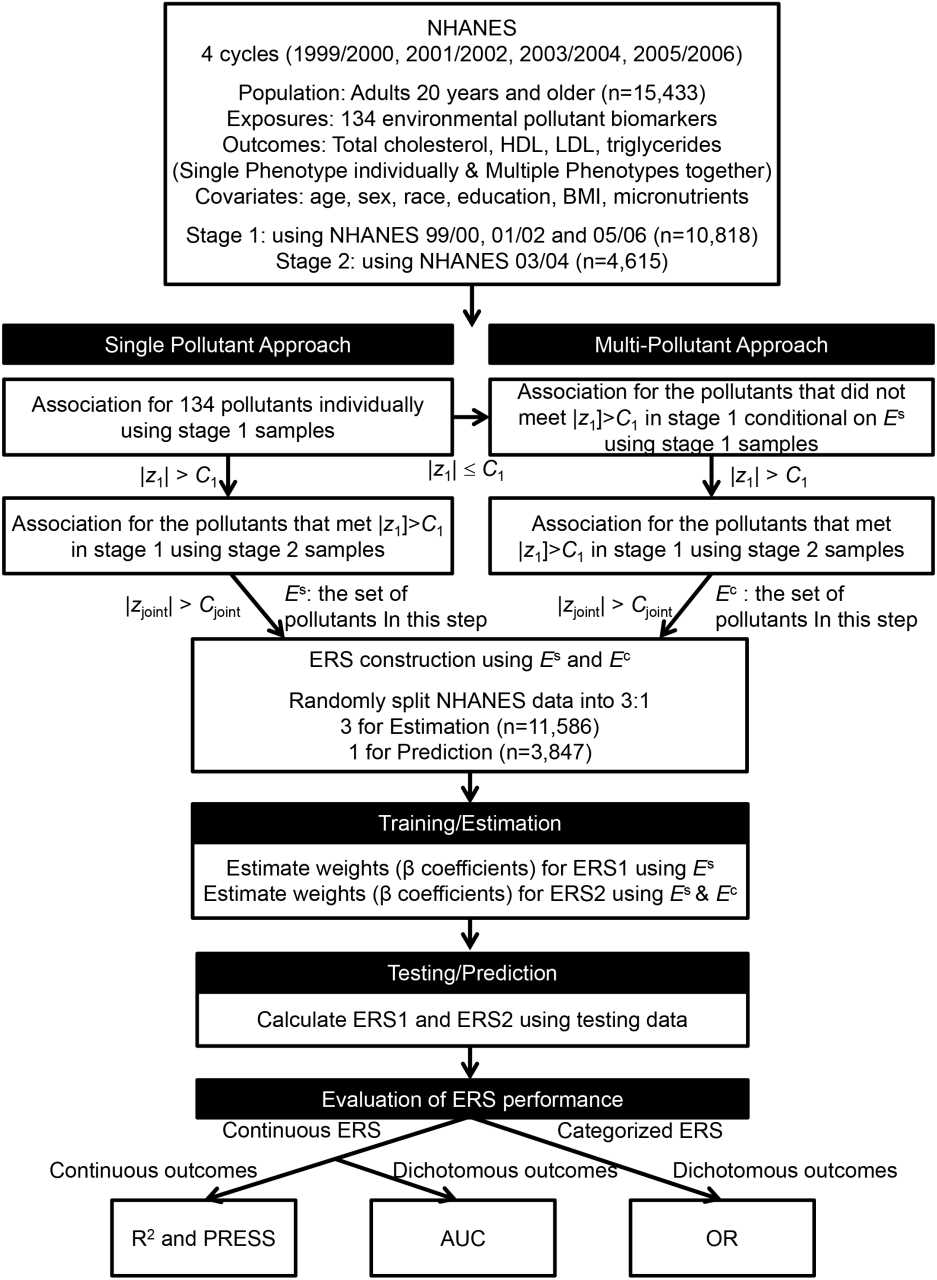
Schematic plot of statistical methods for Environmental Risk Score.

The ERS consists of pollutants selected in the multivariate single- or multi-pollutant analyses. Its construction and assessment steps were the same as in Section 2. A schematic representation of the procedures is presented in Figure 1.

## Results

Table 1 shows population characteristics of the stage 1 and 2 samples. Mean (SD) age and the proportion female were 48 (18.7) years and 53.5% in Stage 1 and 50 (19.5) years and 51.9% in Stage 2, respectively. The mean BMI was 28.4 kg/m^2^ in both Stages. The Stage 1 samples included more Mexican American and other Hispanic and were less educated than the Stage 2 samples. Participants in the Stage 1 had lower HDL (53.0 vs. 54.7 mg/dL) and higher triglycerides (150.2 vs. 140.0 mg/dL) than those in the Stage 2. Total cholesterol was highly correlated with LDL (Spearman correlation coefficient (rho) = 0.86) but modestly correlated with HDL (rho = 0.16) and triglycerides (rho = 0.37) (Table S2). HDL was inversely correlated with triglycerides (rho = –0.42).

**Table 1:** Population characteristics by two stage samples.

Variable	Stage 1 Samples (n = 10818)	Stage 2 Samples (n = 4615)
Continuous (Mean (SD))		
Age (years)	48.0 (18.7)	50.3 (19.5)
BMI (kg/m^2^)	28.4 (6.4)	28.4 (6.3)
Total cholesterol (mg/dL)	201.8 (43.9)	202.0 (44.0)
HDL (mg/dL)	53.0 (16.3)	54.7 (16.3)
LDL (mg/dL)	118.9 (37.8)	119.9 (38.1)
Triglycerides (mg/dL)	150.2 (135)	140.0 (139)
Categorical (*N* (%))		
Gender		
Male	5029 (46.5)	2220 (48.1)
Female	5789 (53.5)	2395 (51.9)
Race/Ethnicity		
Non-Hispanic White	5397 (49.9)	2447 (53.0)
Mexican American	2433 (22.5)	925 (20.0)
Non-Hispanic Black	2121 (19.6)	905 (19.6)
Other Hispanic	498 (4.6)	139 (3.0)
Others	369 (3.4)	199 (4.3)
Education		
< High School	3383 (31.3)	1356 (29.4)
High School	2522 (23.3)	1159 (25.1)
College or Above	4913 (45.4)	2100 (45.5)
Study Year		
1999–2000	3089 (28.5)	-
2001–2002	4736 (43.8)	-
2003–2004	-	4615 (100)
2005–2006	2993 (27.7)	-

HDL, high-density lipoprotein cholesterol; LDL, low-density lipoprotein cholesterol.

Of 31 micronutrient measures in blood, we identified 12 significant predictors for total cholesterol, 9 for HDL, 9 for LDL and 11 for triglycerides (Table S3). Measures of B vitamins (folate, B12, methylmalonic acid), vitamin A (retinol, retinyl palmitate, retinyl stearate), carotenoids (α-carotene, β-carotene, β-cryptoxanthin, lutein/zeaxanthin, lycopene), and/or vitamin E (α- and γ-tocopherol) were selected for each lipid outcome. These phenotype-specific nutrient variables along with the pre-selected base covariates were adjusted for when identifying environmental pollutants for ERS.

### Discovery of Environmental Pollutants for ERS

Table 2 shows environmental pollutants that reached the significance threshold (*C_joint_* of 0.01) for each lipid outcome and their estimated weights (regression coefficients) for ERS from single-pollutant models (ERS1) and a multi-pollutant model (ERS2). Figure S1 presents visual distributions of the P values for the individual environmental pollutants examined in the Stage-1 samples (Manhattan plot [41]). Out of 134 environmental pollutants, 11, 9, 5 and 23 pollutants were significantly associated with total cholesterol, HDL, LDL, and triglycerides, respectively, in single pollutant models (marginal analyses) with adjustment for the base covariates and phenotype-specific nutrients. Note that the weights in Table 2 are the regression coefficients for each log-transformed exposure in relation to the log-transformed lipid outcome, which are not directly interpretable. Generally, percent changes for a two-fold increase in exposure concentrations are presented as [exp(regression coefficient×log(2)) –1]×100%. For example, a two-fold increase in blood lead was associated with a 19% higher levels of total cholesterol ([exp(1.71×log(2)) –1]×100%  = 19%). Since we used these weights to construct ERS rather than interpret the associations of individual pollutants, we presented the direct weights rather than more interpretable estimates (percent changes). Also note that less significant associations in ERS2 compared with ERS1 are mainly due to lower power due to fitting of a larger model with larger number of parameters and with multiple pollutants that are potentially correlated. Two pollutants (1,2,3,4,6,7,8-HpCDD and PCB 177) for total cholesterol and 4 pollutants (PCB 118, PCB 138, PCB 153 and 3,3,4,4,5,5-PnCB) for triglycerides were additionally identified in conditional analyses in which the pollutants selected in the previous two-stage analyses were included as covariates. No further pollutants were identified in relation to HDL and LDL in the conditional analyses. Therefore, a total of 13 pollutants for total cholesterol, 9 for HDL, 5 for LDL and 27 for triglycerides were identified and used to construct ERS for each outcome. Various persistent organic pollutants (POPs) were positively associated with total cholesterol and triglycerides and inversely associated with HDL in single-pollutant models but the association directions for some POPs (2,3,4,7,8-PnCDF, 3,3,4,4,5-HxCB, PCB 138, PCB 146, PCB 156, PCB 177, PCB 180, and PCB 183) changed in the multi-pollutant model, probably due to multi-collinearity. Phthalates were inversely associated with HDL. Cadmium and lead were associated with lipid outcomes in expected directions, that is, higher concentrations of cadmium and lead were associated with higher levels of lipid outcomes except the association between lead and HDL (good cholesterol) which was positive. Interestingly, the mercury (blood total and urinary) and arsenobetaine measures were inversely associated with triglycerides; as were perfluoroheptanoic acid and diethylphosphate with LDL.

**Table 2:** Estimated environmental risk score (ERS) weights for environmental pollutants selected for each phenotype.

Class	Variable name in NHANES	Pollutant Name	Weight^a^ (10^−2^)							
			Total cholesterol		HDL		LDL		Triglyceride	
			ERS1^b^	ERS2^c^	ERS1^b^	ERS2^c^	ERS1^b^	ERS2^c^	ERS1^b^	ERS2^c^
Heavy metals	LBXBPB	Lead in blood	1.71#	1.36#	1.62#	1.95#	2.54#	2.31#		
	LBXBCD	Cadmium in blood	1.18#	0.84#					4.69#	4.73#
	URXUCD	Cadmium in urine			–1.32#	–1.22#	0.98^∧^	0.78		
	LBXTHG	Total mercury in blood							–2.95#	–1.65*
	URXUHG	Mercury in urine							–2.15#	–1.58#
	URXUAB	Arsenobetaine in urine							–0.93#	–0.51^∧^
	URXUSB	Antimony in urine			–1.23#	–0.43^∧^				
Phthalates	URXMZP	Mono-benzyl phthalate			–0.62#	–0.09				
	URXMIB	Mono-isobutyl phthalate			–0.80#	–0.33				
	URXMBP	Mono-n-butyl phthalate			–0.75#	–0.09				
	URXMC1	Mono-(3-carboxylpropyl) phthalate			–0.70*	–0.17				
PAHs	URXP07	2-phenanthrene							1.41#	1.32#
PFCs	LBXPFHP	Perfluoroheptanoic acid					–3.99*	–3.84*		
Dioxins and Furans	LBXTCD	2,3,7,8-TCDD	0.64^∧^	0.51^∧^			1.55^∧^	1.49^∧^		
	LBXF03	2,3,4,7,8-PnCDF							1.72*	–0.24
	LBXF07	2,3,4,6,7,8-HxCDF							5.18#	4.71#
	LBXF08	1,2,3,4,6,7,8-HpCDF	0.82#	0.75*						
Dioxin-like PCBs	LBX066	PCB 066							2.44^∧^	2.12^∧^
	LBX105	PCB 105							2.05*	0.96
	LBX118	PCB 118							1.79#	0.34
	LBX156	PCB 156	0.54*	–0.36					1.59*	–0.90
	LBXPCB	3,3,4,4,5,5-PnCB							1.57#	0.70
	LBXHXC	3,3,4,4,5-HxCB	0.61*	–0.17					2.71#	2.15^∧^
Non-dioxin-like PCBs	LBX099	PCB 099							1.76*	1.82
	LBX138	PCB 138							1.26^∧^	–2.48
	LBX146	PCB 146	0.56^∧^	–0.12					1.68^∧^	–0.13
	LBX153	PCB 153							1.31^∧^	1.41
	LBX156	PCB 156	0.54*	–0.36					1.59*	–0.90
	LBX170	PCB 170	0.79#	0.75					2.39#	3.36
	LBX177	PCB 177	0.46^∧^	0.19					0.78	–1.41
	LBX180	PCB 180	0.69#	0.42					2.00#	–3.45
	LBX183	PCB 183	0.48^∧^	0.07					0.88	–1.27
	LBX187	PCB 187	0.69#	0.05					2.34#	2.41
Organo-chlorine pesticides	LBXPDT	*p*,*p*-DDT							1.74#	0.78
	LBXOXY	Oxychlordane							2.64#	1.53*
	LBXHPE	Heptachlor Epoxide			–1.36#	–0.98^∧^			3.18#	1.93^∧^
	LBXDIE	Dieldrin			–1.36#	–0.58			3.03#	0.78
Dialkyl metabolites	URXOP2	Diethylphosphate					–0.35	–0.34		
Total number identified			13		9		5		27	

HDL, high-density lipoprotein cholesterol; LDL, low-density lipoprotein cholesterol; PAHs, polycyclic aromatic hydrocarbons; PFCs, perfluorinated compounds; PCBs, polychlorinated biphenyls; TCDD, tetrachlorodibenzodioxin; PnCDF, pentachlorodibenzofuran; HxCDF, hexachlorodibenzofuran; HpCDF, heptachlorodibenzofuran; PnCB, pentachlorobiphenyl; HxCB, hexachlorobiphenyl; DDT, dichlorodiphenyltrichloroethane.

All models were adjusted for age, gender, race/ethnicity, education, BMI and phenotype-specific nutrients shown in Table S3.

a Weights were estimated using the training data (n = 11586).

b ERS constructed with coefficient estimates from single-pollutant models as weights.

c ERS constructed with coefficient estimates from multi-pollutant models as weights.

# *p*-value<0.001,

*0.001≤*p*-value<0.01, and ^∧^0.01≤*p*-value<0.05.

### Risk Prediction by ERS and its Associations with Lipid Outcomes

The ERS’s from single-pollutant models ranged from −0.068 to 0.239 (mean±SD = 0.090±0.043) for total cholesterol (fit as a continuous outcome (log-transformation). Same for other outcomes); −0.226 to 0.205 (0.030±0.057) for HDL; −0.059 to 0.195 (0.088±0.029) for LDL; and −1.278 to 0.563 (–0.445±0.228) for triglycerides. Those from a multi-pollutant model ranged from −0.009 to 0.135 (0.058±0.019) for total cholesterol; −0.013 to 0.152 (0.061±0.022) for HDL; −0.054 to 0.183 (0.086±0.027) for LDL; and −0.291 to 0.339 (–0.009±0.082) for triglycerides (Table S4). The ERS2 were generally smaller than the ERS1 because of more inverse associations in ERS2.

Table 3 presents risk prediction measures by ERS when outcomes were continuous (R^2^ and PRESS) and dichotomized (AUC). Base covariates and micronutrients explained approximately 13% of the variation for LDL, 26% for HDL, 33% for total cholesterol and 37% for triglycerides. ERS constructed with coefficients from single-pollutant models (ERS1) additionally explained variations from 0.33% for LDL to 0.72% for triglycerides. Addition of ERS1 decreased the PRESS by from 0.33% [(539.62–537.84)/539.62] for LDL to 1.1% [(967.24–956.76)/967.24] for triglycerides. When the dichotomous outcomes were used, the addition of the ERS1 only minimally modestly improved the AUC for each lipid outcome (Table 3 and Figure S2). Similar results were found with the ERS constructed with coefficients from multi-pollutant models (ERS2). Similar risk predictions were observed in the multi-phenotype approach although six new pollutants were identified in the multi-phenotype approach (Table S5).

**Table 3:** Risk prediction by continuous environmental risk score (ERS) using single-phenotype approach^a^ (n = 3847).

Phenotype	Continuous Outcome						Dichotomized^b^ Outcome		
	Model 1^c^		ERS1^d^		ERS2^e^		Model 1^c^	ERS1^d^	ERS2^e^
	R^2^	PRESS^f^	R^2^	PRESS^f^	R^2^	PRESS^f^	AUC^g^	AUC^g^	AUC^g^
Total cholesterol	0.3270	122.46	0.3306	121.88	0.3308	121.85	0.7672 (0.7523, 0.7820)	0.7695 (0.7547, 0.7842)	0.7691 (0.7543, 0.7838)
HDL	0.2636	231.70	0.2677	230.52	0.2665	230.91	0.7193 (0.7024, 0.7362)	0.7217 (0.7050, 0.7385)	0.7208 (0.7040, 0.7376)
LDL	0.1342	539.62	0.1375	537.84	0.1376	537.80	0.7213 (0.7050, 0.7376)	0.7255 (0.7093, 0.7416)	0.7253 (0.7091, 0.7414)
Triglyceride	0.3709	967.24	0.3781	956.76	0.3775	957.70	0.8164 (0.8021, 0.8306)	0.8178 (0.8036, 0.8320)	0.8183 (0.8041, 0.8324)

a Pollutants selected by single-phenotype regression (n = 13, 9, 5 and 27 for total cholesterol, HDL, LDL and triglyceride, respectively) to construct ERS which was computed in the validation data (n = 3847), with adjustment for base covariates and phenotype-specific micronutrients.

b Continuous phenotypes dichotomized to be high vs. low by thresholds: 200 mg/dL for CHOL, 40 mg/dL (male) or 50 mg/dL (female) for HDL, 130 mg/dL for LDL and 150 mg/dL for TRIG.

c adjusted for base covariates and phenotype-specific micronutrients.

d Model 1 plus ERS constructed with coefficient estimates from single-pollutant models as weights.

e Model 1 plus ERS constructed with coefficient estimates from multi-pollutant models as weights.

f Predicted residual sums of squares.

g Area under the receiver operating characteristic (ROC) curve and its 95% confidence interval computed with 2000 stratified bootstrap replicates.

Table 4 shows ORs of having adverse levels of lipid outcomes comparing the highest vs. the lowest quintiles of ERS. After controlling for base covariates and micronutrients, ORs of total cholesterol comparing the highest vs. the lowest quintiles were from 1.45 (95% confidence interval (CI), 1.11, 1.89) for ERS1 and single-phenotype approach to 1.78 (95% CI, 1.34, 2.37) for ERS1 and multi-phenotype approach. For HDL, ORs ranged from 1.37 (95% CI, 1.08, 1.75) for ERS1 and single-phenotype approach to 1.57 (95% CI, 1.23, 1.99) for ERS2 and multi-phenotype approach. For LDL, the highest quintile had a 82% higher odds of having high LDL levels (95% CI, 1.39, 2.38) compared with the lowest quintile in single-phenotype approaches, whereas the associations were relatively weak in multi-phenotype approaches (OR = 1.36 (95% CI, 1.06, 1.74) for ERS1 and 1.26 (95% CI, 0.97, 1.64) for ERS2). For triglycerides, ORs ranged from 1.54 (95% CI, 1.15, 2.06) for ERS2 and single-phenotype approach to 2.03 (95% CI, 1.52, 2.70) for ERS2 and the multi-phenotype. These ORs were comparable to or even stronger than those for socio-demographic factors or BMI (Table S6). For example, the OR of the association between total cholesterol and ERS from single-pollutant models (1.45) was consistent with ORs for females vs. males (1.47); for non-Hispanic blacks vs. non-Hispanic white (1.42); and for a 30 kg/m^2^ increase in BMI (1.47); and stronger than ORs for <high school vs. college or higher (1.20).

**Table 4:** Odds ratios (95% CIs) for environmental risk score (ERS) categorized by quintile^a^ (n = 3847).

Phenotype^b^	Single-phenotype Approach^c^		Multi-phenotype Approach^d^	
	ERS1^e^	ERS2^f^	ERS1^e^	ERS2^f^
Total cholesterol	1.450 (1.112, 1.892)	1.722 (1.317, 2.252)	1.781 (1.337, 2.374)	1.564 (1.191, 2.054)
HDL	1.372 (1.077, 1.748)	1.450 (1.144, 1.838)	1.471 (1.142, 1.894)	1.565 (1.230, 1.990)
LDL	1.824 (1.394, 2.386)	1.820 (1.391, 2.381)	1.357 (1.061, 1.735)	1.262 (0.973, 1.637)
Triglyceride	1.843 (1.366, 2.487)	1.536 (1.147, 2.056)	1.758 (1.275, 2.424)	2.027 (1.521, 2.703)

a Odds ratios for dichotomized phenotype (high vs. low) comparing subjects with ERS in the top 20% to those in the bottom 20%, adjusted for covariates and micronutrients.

b Dichotomization thresholds: 200 mg/dL for total cholesterol, 40 mg/dL (male) or 50 mg/dL (female) for HDL, 130 mg/dL for LDL and 150 mg/dL for triglyceride.

c Pollutants selected by single-phenotype regression (n = 13, 9, 5 and 27 for total cholesterol, HDL, LDL and triglyceride, respectively) to construct ERS, adjusted for phenotype-specific micronutrients.

d Pollutants selected by multi-phenotype regression (n = 45) to construct ERS, adjusted for union of selected micronutrients (n = 14).

e ERS constructed with coefficient estimates from single-pollutant models as weights.

f ERS constructed with coefficient estimates from multi-pollutant models as weights.

**Figure 2:**
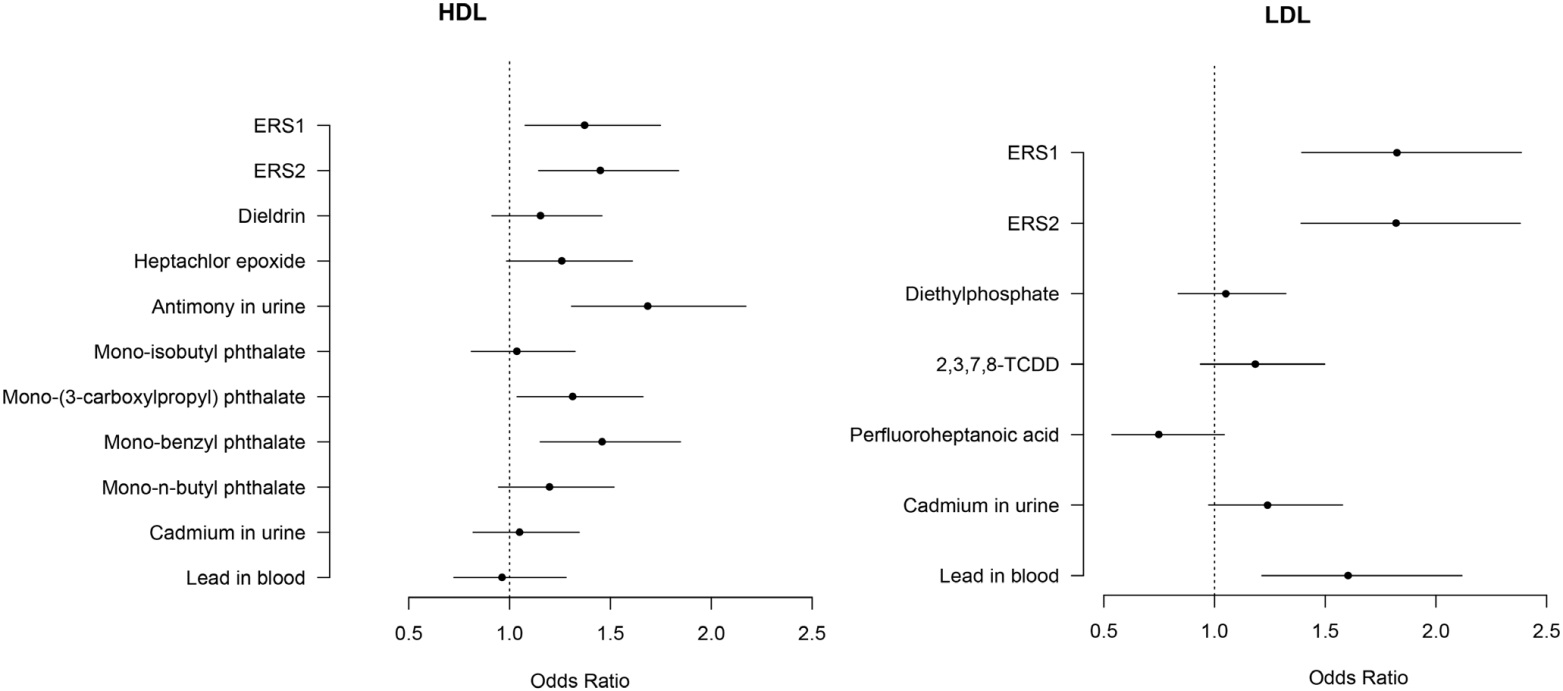
Odds ratios (95% confidence intervals) of having adverse levels of HDL (40 mg/dL for men and 50 mg/dL for women) and LDL (130 mg/dL) comparing the highest vs. the lowest quintiles of ERS and individual pollutants that compose the ERS. Models were adjusted for age, gender, race/ethnicity, education, BMI, and phenotype-specific micronutrients.

Figure 2 shows ORs of having adverse levels of HDL and LDL for individual pollutants that compose the ERS. Three out of the 9 pollutants (antimony, mono-benzyl phthalate, mono-(3-carboxylpropyl) phthalate) had significant positive associations with the odds of HDL, the rest except for blood lead had weak non-significant positive associations and blood lead had a weak non-significant inverse association. One of the 5 pollutants (blood lead) had a significant positive association with the odds of LDL and the rest had weak non-significant associations. In particular, the effect sizes of ERS’s in relation to LDL were larger than any of the effect sizes of individual pollutants. Here we present ORs of HDL and LDL because their ERS’s comprise the smaller number of pollutants (9 and 5 pollutants each). The plots for total cholesterol and triglycerides are shown in Figure S3.

## Discussion

In this study, we propose an Environmental Risk Score (ERS) as a novel approach that integrates information on the health effects of multiple pollutant exposures. We used serum lipid measures and various classes of pollutant biomonitoring data from NHANES to illustrate and validate this approach. Important environmental risk factors for lipid outcomes were identified individually (single-phenotype approach) or together (multi-phenotype approach) while controlling for socio-demographic risk factors and nutrients. Although the contributions of ERS to overall risk predictions for lipid outcomes (i.e., R^2^, PRESS and AUC) were modest after accounting for important socio-demographic factors and nutrients, we found relatively stronger associations between ERS and lipid outcomes than with individual pollutants. The magnitudes of the observed associations between ERS and lipid outcomes were comparable to or stronger than those for socio-demographic factors or BMI.

Although the importance of evaluating the health effects of multi-pollutant exposures has recently been recognized [18], [42], only a few studies have been conducted, mostly focused on multiple air pollutants [10], [21], [43]–[46], probably due to methodological challenges, such as collinearity, measurement errors, potential interaction between pollutants and potential non-linear exposure-health relationships [16]. Patel et al. adopted newer techniques used in genomics and proposed an Environment-Wide Association Study (EWAS) [5], [6]. This approach provided excellent insight to identify ‘top hit’ pollutants. However, few epidemiologic studies have provided methods to estimate combined effects or to predict risks from multi-pollutant exposure [43], [47].

Hong et al. examined the combined effects of 4 air pollutants (particulate matter<10 μm (PM_10_), nitrogen dioxide (NO_2_), sulfur dioxide (SO_2_), and ozone) by summing each pollutant concentration divided by its mean (i.e., relative concentrations) and then fitting this index as an independent variable [47]. They found that the combined index had a stronger association with mortality than individual pollutants. In a study of indoor exposure to volatile organic compounds (VOCs) and respiratory health, Billionnet et al. computed a global VOC score of 20 VOCs by dichotomizing individual VOC as 1 if greater than the 75^th^ percentile and otherwise 0 and then summing the 20 dichotomous VOCs, which indicates the number of VOCs whose concentrations were relatively high within the study population (range 0–17) [43]. Each additional VOC with a concentration higher than the 75^th^ percentile was associated with 7% (95% CI, 1.00–1.13) and 4% (95% CI, 1.00–1.08) higher odds of asthma and rhinitis, respectively. Although these studies evaluated the combined effects of multi-pollutants, their approaches did not account for the relative effects of individual pollutants on the phenotype of interest, that is, each pollutant was not weighted depending on its relative effect size. Our study aimed to obtain a more precise relative effect size of each pollutant on each lipid outcome by estimating the weights (regression coefficients) from a randomly split training dataset and then computed ERS in an independent validation dataset.

In the real-world, we are exposed to multiple pollutants which may contribute to disease susceptibility in combination or as mixtures. In contrast, individual pollutants may have relatively small effects. Our study supports this notion that only a few pollutants were significantly associated with serum lipids levels while many individual pollutants had relatively weak associations (Figure 2 and Figure S3). The ERS as a multi-pollutant approach allows us to integrate those relatively small effects from multiple pollutants and provides a better opportunity to identify subpopulations that are at higher risk for diseases. We used multi-pollutant information at different steps of our process. Our discovery approach is different from Patel’s [5], [6] as we performed analysis with single pollutant models and then evaluated additional pollutants conditional on the identified pollutants. We then formed ERS using the set of all pollutants identified via this process using the weights from assessing them one at a time (ERS1) and jointly (ERS2). It appears that in terms of overall prediction, ERS1 and ERS2 were very similar in performance (Table 3), however, ERS2 was often slightly better in terms of risk stratification (Table 4). It is not possible to conclude definitively, without extensive and exhaustive simulation studies, which one performs better. Also, one could modify ERS2 by filtering potentially correlated predictors through variable selection, and reducing its variability. Although high risk groups were identified by the ERS in the present study, the ERS showed only modest improvement in lipid-related risk prediction of above and beyond the effect of traditional risk factors including sociodemographic and dietary factors (e.g., AUC improvements of 0.72 to 0.82, Table 3 and Table S5). This finding may not be surprising because a marker with an OR of 3 or lower is usually a poor tool for classifying or predicting risk for individuals [48]. In fact, the improvements of risk prediction/classification by the ERS are similar to the AUC improvements for coronary heart disease risk prediction by genetic risk scores (GRS) found in the Atherosclerosis Risk in Communities (ARIC) (from 0.742 to 0.749), Rotterdam Study (from 0.729 to 0.734) and Framingham Offspring Study (from 0.773 to 0.775) [49]. We also point out that for GWAS studies, a polygenic risk score has also contributed very modestly to risk prediction as measured by increment in AUC or R^2^, however, similar risk stratification properties across the quintiles of genetic risk scores have been noted [23]. Nonetheless, our findings imply that ERS can better determine potential risk stratification where individuals are at increased risk of high lipid levels and related cardio-metabolic diseases than single pollutant approaches. The proposed ERS may allow us to identify susceptible subpopulations where targeted interventions are necessary and could have the greatest benefits [27].

Theoretically, a multiple phenotype approach always reduces the number of tests that are conducted, and also increases power by exploiting correlation across phenotypes. In our study, we discovered that the multi-phenotype approach leads to elevated ORs in Table 4, aiding with risk stratification. In general, if there is correlation among the pollutants, the discovery approach based on conditional associations may yield new results. If there is correlation among the outcomes or different phenotypes, the multi-phenotype approach, in spite of being a test with higher degrees of freedom, will yield a more powerful analysis. For example, six new pollutants were discovered with the multi-phenotype approach in our case study.

Our study has numerous limitations. The individual pollutants used to construct the ERS were identified in linear regression models with log-transformation due to skewed distributions, which assumes linear (in fact, log-linear) exposure-outcome relationships for all individual pollutants. However, not all pollutants are linearly associated with health outcomes, for example, some pesticides and/or other endocrine disrupting chemicals may have thresholds or non-monotonic dose-responses [50], [51]. Pollutants whose dose-responses were misspecified may not be selected and not contribute to the ERS. Examining non-linearity in each of the single pollutant models may identify new pollutants but construction of a simple weighted risk score like ERS would no longer be possible, which led us to a linear regression based screening strategy in this initial paper. Moreover the ERS itself may have a non-linear association with the outcome when treated as a single predictor. We used quintiles of ERS to somewhat address this issue in the association models but a completely flexible generalized additive model will be more appropriate from a statistical point of view. We tried to retain simplicity in our approach for usability and thus compromised on some finer points that may be expanded upon in the future.

We did not consider pollutant-pollutant or pollutant-nutrient interactions when important individual pollutants were selected. Some pollutants may interact and have synergy. A well-known example is cigarette smoking and asbestos on lung cancer [52], [53]. On the other hand, beneficial nutrients may mitigate toxic effects of pollutants. For example, people with higher intake of antioxidant vitamins, B-vitamins (folate and vitamin B12) or omega-3 fatty acids had lower effects of air pollution [54]–[56]. Conventional statistical approach that includes cross-product terms of two interacting factors may have low power and therefore effect estimates would be unstable. A recent study by Sun et al. [10] proposed statistical strategies to examine multi-pollutants and their interactions using a two-stage model. Other dimension reduction techniques may also work for estimating risk models when a large number of pollutants and their interactions exist. A planned future study accounting for pollutant-pollutant and pollutant-nutrient interactions is expected to improve the model prediction, and therefore, potentially the utility of ERS.

We used an arbitrary significance level of 0.01 to account for false positive rate. One reason is that we wanted to allow environmental pollutants that had even modest associations to be included in the ERS. We conducted sensitivity analyses using significance levels of 0.05 and 0.001 and applied these different thresholds to the AUC as shown in Table 3. Under the significance level of 0.05, 30 pollutants (vs. 13 under the significance of 0.01) for total cholesterol; 16 (vs. 9) for HDL; 5 (vs. 5) for LDL; and 34 (vs. 27) for triglycerides were identified. However, the improvement in the AUC and OR were minimal. Using a significance level of 0.001, the number of pollutants identified decreased substantially, especially for LDL. The decrease in AUC was mainly for LDL while the decrease in OR was found for all phenotypes. Therefore we chose the intermediate threshold of 0.01. Even higher significance levels (e.g., alpha of 0.1) have been used as “pruning criteria” in genetic risk scores [57], [58], therefore, genetic markers conferring only modest levels of disease risk could be aggregated in the risk score. In general, a liberal threshold is often noted to perform better for prediction as compared to controlling false discovery rate for identification of variables [59].

Although we include many environmental pollutants that are widespread and available in NHANES, we were not able to account for *all* environmental pollutants as it is unrealistic, the data were not available and not all environmental pollutants have been identified as yet. Also, we limited our analysis to chemical environmental pollutants in constructing the ERS. Recently, a new concept of the exposome, that is, the totality of exposures over the course of a lifetime, has been proposed [60]–[65] and the need for more complete *non-genetic* exposure assessment in epidemiologic research has been emerging, as emphasized in the strategic themes defined by the National Institute of Environmental Health Sciences (NIEHS) (http://www.niehs.nih.gov/about/strategicplan/). Our proposed approach will be useful to identify important individual factors and to combine their risks, which eventually will advance our understanding of health responses to the complex nature of multi-pollutant exposures.

Each individual pollutant has different degrees of measurement error. Exposure measurement errors are generally non-differential when the errors are independent of each other and the disease status [66]. Therefore, it is expected that environmental pollutants measured with less non-differential measurement error such as those with lower temporal variability are more likely to be detected (e.g., PCBs vs. phthalates). However, differential measurement errors may occur when exposure measurement errors are not independent because some of the effects of more poorly measured exposures may be transferred to the effect estimates of better-measured exposures [67]. In addition, most of the pollutant variables used in our study are subject to a limit of detection (LOD). Several *ad hoc* substitution methods, such as substitution of LOD/2 or LOD/√2 for values below LOD, are widely used (NHANES used LOD/√2). These *ad hoc* methods, however, can lead to bias especially when the proportion of values below LOD is high [68]. Maximum likelihood estimation based on a parametric joint distribution assumption for all the exposures, for example, multivariate normal distribution, may reduce potential bias if the parametric distribution assumption is correct [69].

Exposure data were collected cross-sectionally at one point in time, yet exposures are subject to temporal variation. This issue becomes particularly important when examining health effects of non-persistent short-lived environmental pollutants, such as BPA and phthalates. A recent study of urinary BPA and type-2 diabetes using three NHANES cycles found a significant association which was confirmed in one cycle (2003–2004) but not in the other two cycles. This finding indicates possible exposure misclassification due to a single urine sample [70]. Reliable exposure biomarker data assessed based on repeatedly collected samples is warranted to reduce exposure misclassification.

We did not consider differential risk prediction in different subpopulations. Emerging evidence suggests that certain subgroups may be more responsive to environmental pollutant exposure. Women are known to take up more divalent metals such as lead and cadmium due to iron depletion [71]. Stronger associations between lead and hypertension have been found in some racial/ethnic populations [72], [73]. Sex- or race/ethnic group-specific biological differences, such as differences in body iron and estrogen levels between men and women, or socially determined gender- or race/ethnic group differences, such as different psychosocial stress levels, may confer susceptibility to health responses to pollutant exposures [74], [75]. Sex-specific or race/ethnic group-specific ERS’s may provide better risk prediction as well as risk assessment.

Our results may be biased due to residual confounding. Urinary creatinine adjustment has been recommended for urinary biomarkers to correct for dilutions of pollutant concentrations in spot urine samples [76]. The main purpose of the present study is to introduce a novel ERS approach as a proof of concept illustration rather than to identify potential environmental factors related to health outcomes and estimate the associations as done in previous EWAS. Variance may be somewhat underestimated and the observed findings may not be generalizable to the US population.

Because not all environmental pollutants were measured in the entire population, we imputed unmeasured or missing pollutant data to maximize the power. We used a single imputation because our main goal was to introduce the approach of ERS, but multiple imputations after taking the uncertainty in imputed values into account would be a more appropriate approach. Imputation may be necessary for meta-analyses of multiple ERS studies in the future because it is unlikely that every cohort has a uniform set of pollutants measured. Careful data harmonization and imputation may increase the power of the analysis if correlated exposures and covariates are observed in one cohort that are predictive of exposures in another cohort where those exposures are missing. However, the imputation issue will merit a complete paper in its own right, as imputation with high dimensional data is still very much an evolving topic in statistical research [77]. In summary, the present study suggests ERS is a promising tool for integrating disease risks from multi-pollutant mixture exposures. The ERS is a simplest form of data reduction, characterizing the summary exposure burden like a polygenic risk score in genetics [27]. This new approach supports the need for moving from a single-pollutant to a multi-pollutant framework for new discoveries and better risk stratification. Combining information from ERS along with known predictors can improve disease prediction. Also, the ERS along with genetic risk score can potentially provide a way to reduce dimension and increase the power in studies of gene-environment interaction. More generally, ERS can be taken as a measure of summary/background burden of environmental exposure and it will be interesting to explore whether the effect of a certain gene, behavioral factors (diet, physical activity, smoking) or another pollutant is larger if individuals are in the highest quartile of ERS. The contribution of ERS to risk prediction and classification warrants further studies.

Data Sharing: The data and codes used for illustration of our approach are available at http://www-personal.umich.edu/bhramar/software/.

## Supporting Information

Figure S1 **Manhattan plots representing the P value distributions of the individual environmental pollutants examined using the stage 1 samples.** Y-axis indicates –log10(p-value) of the regression coefficient for each of the environmental pollutants, adjusted for age, gender, race/ethnicity, education, body mass index and phenotype-specific micronutrients. The horizontal dotted line represents the p-value of 0.01. X-axis indicates 13 classes of environmental pollutants: 1) heavy metals; 2) phthalates; 3) environmental phenols; 4) polycyclic aromatic hydrocarbons (PAHs); 5) volatile organic compounds (VOCs); 6) perfluorinated compounds (PFCs); 7) dioxins and furans; 8) dioxin-like polychlorinated biphenyls (PCBs); 9) non-dioxin-like PCBs; 10) organochlorine pesticides; 11) organophosphate dialkyl metabolites; 12) herbicides; and 13) pesticides phenols. Each color represents one class.
(PDF)

Figure S2 **Receiver operating characteristic (ROC) curves for four phenotypes.** The dotted line denotes the null curve. The black curve is for the model with only covariates. The blue curve is for the model with both covariates and phenotype-specific micronutrients. The red curve is for the model with environmental risk score (ERS), covariates and phenotype-specific micronutrients.
(PDF)

Figure S3 **Odds ratios (95% confidence intervals) of having adverse levels of total cholesterol (CHOL: 200 mg/dL) and triglyceride (TRIG: 150 mg/dL) comparing the highest vs. the lowest quintiles of ERS and individual pollutants that compose the ERS.** Models were adjusted for age, gender, race/ethnicity, education, BMI, and phenotype-specific micronutrients.
(PDF)

Table S1 **Environmental pollutants evaluated in the present study (n = 134).**
(PDF)

Table S2 **Spearman correlation coefficients between four phenotypes.**
(PDF)

Table S3 **Micronutrients selected for each phenotype using Bayesian model averaging (BMA).**
(PDF)

Table S4 **Distributions of Environmental Risk Scores (ERS) (n = 3847).**
(PDF)

Table S5 **Risk prediction by continuous environmental risk score (ERS) using multi-phenotype approach^a^ (n = 3847).**
(PDF)

Table S6 **Regression outputs for each lipid outcome in relation to ERS1.**
(PDF)

File S1 **Diagnostic Analysis for the Imputation.**
(PDF)
