# Supplementary material for: Environmental Risk Score as a New Tool to Examine Multi-Pollutants in Epidemiologic Research: An Example from the NHANES Study Using Serum Lipid Levels
Source: PLoS One. 2014 Jun 5;9(6):e98632. doi: 10.1371/journal.pone.0098632 (PMC4047033; doi:10.1371/journal.pone.0098632)
Supplement: Figure S1 — Manhattan plots representing the P value distributions of the individual environmental pollutants examined using the stage 1 samples. Y-axis indicates –log10(p-value) of the regression coefficient for each of the environmental pollutants, adjusted for age, gender, race/ethnicity, education, body mass index and phenotype-specific micronutrients. The horizontal dotted line represents the p-value of 0.01. X-axis indicates 13 classes of environmental pollutants: 1) heavy metals; 2) phthalates; 3) environmental phenols; 4) polycyclic aromatic hydrocarbons (PAHs); 5) volatile organic compounds (VOCs); 6) perfluorinated compounds (PFCs); 7) dioxins and furans; 8) dioxin-like polychlorinated biphenyls (PCBs); 9) non-dioxin-like PCBs; 10) organochlorine pesticides; 11) organophosphate dialkyl metabolites; 12) herbicides; and 13) pesticides phenols. Each color represents one class. (PDF) [file pone.0098632.s001.pdf]

Environmental Risk Score as a new tool to examine multi-pollutants in epidemiologic research: an example from the NHANES study using serum lipid levels

Sung Kyun Park, Yebin Tao, John D. Meeker, Siobán D. Harlow, Bhramar Mukherjee

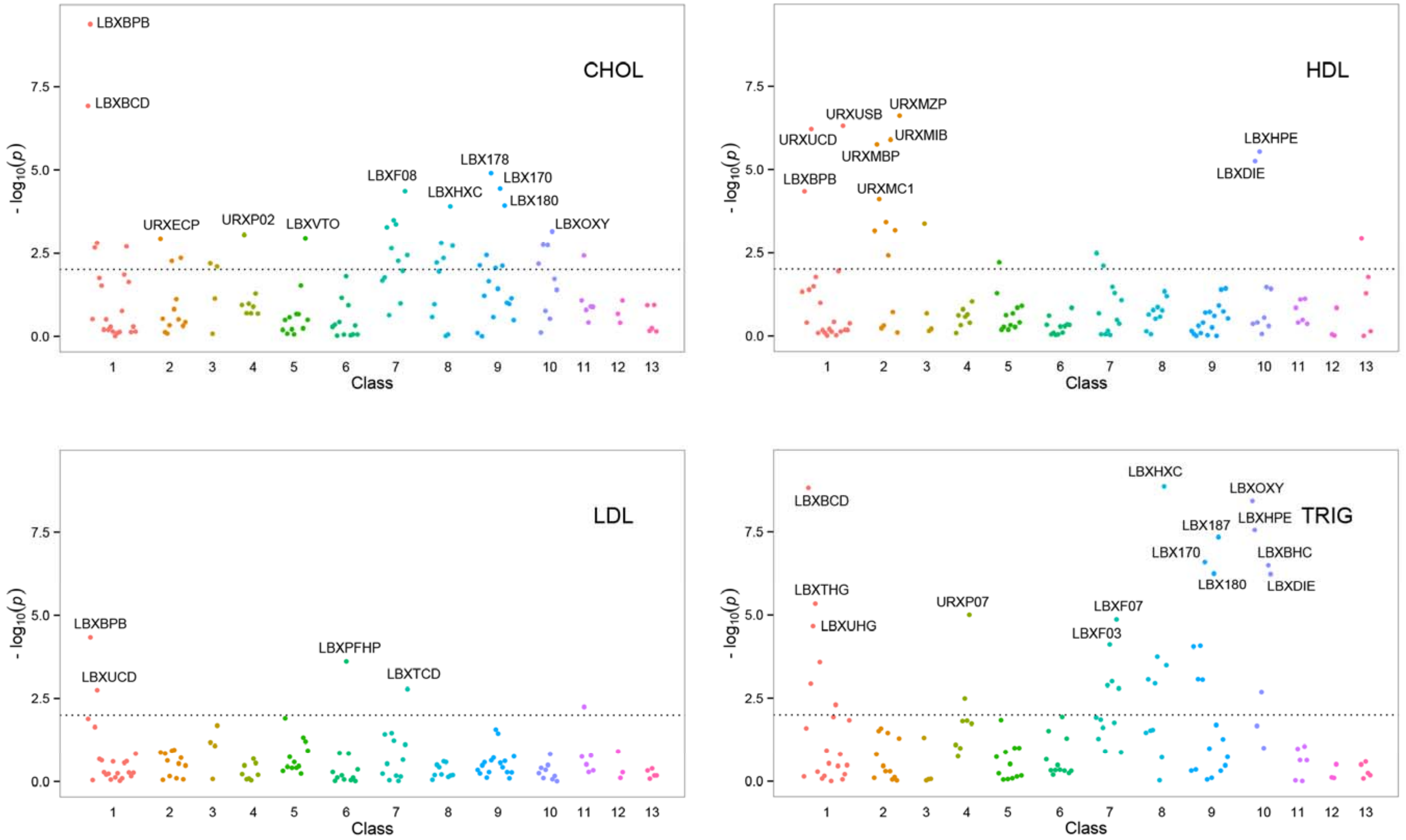

Figure S1. Manhattan plots representing the P value distributions of the individual environmental pollutants examined using the stage 1 samples. Y-axis indicates -log10(p-value) of the regression coefficient for each of the environmental pollutants, adjusted for age, gender, race/ethnicity, education, body mass index and phenotype-specific micronutrients. The horizontal dotted line represents the p-value of 0.01. X-axis indicates 13 classes of environmental pollutants: 1) heavy metals; 2) phthalates; 3) environmental phenols; 4) polycyclic aromatic hydrocarbons (PAHs); 5) volatile organic compounds (VOCs); 6) perfluorinated compounds (PFCs); 7) dioxins and furans; 8) dioxin-like polychlorinated biphenyls (PCBs); 9) non-dioxin-like PCBs; 10) organochlorine pesticides; 11) organophosphate dialkyl metabolites; 12) herbicides; and 13) pesticides phenols. Each color represents one class.
