# Supplementary material for: Environmental Risk Score as a New Tool to Examine Multi-Pollutants in Epidemiologic Research: An Example from the NHANES Study Using Serum Lipid Levels
Source: PLoS One. 2014 Jun 5;9(6):e98632. doi: 10.1371/journal.pone.0098632 (PMC4047033; doi:10.1371/journal.pone.0098632)
Supplement: Figure S2 — Receiver operating characteristic (ROC) curves for four phenotypes. The dotted line denotes the null curve. The black curve is for the model with only covariates. The blue curve is for the model with both covariates and phenotype-specific micronutrients. The red curve is for the model with environmental risk score (ERS), covariates and phenotype-specific micronutrients. (PDF) [file pone.0098632.s002.pdf]

Environmental Risk Score as a new tool to examine multi-pollutants in epidemiologic research: an example from the NHANES study using serum lipid levels

Sung Kyun Park, Yebin Tao, John D. Meeker, Siobán D. Harlow, Bhramar Mukherjee

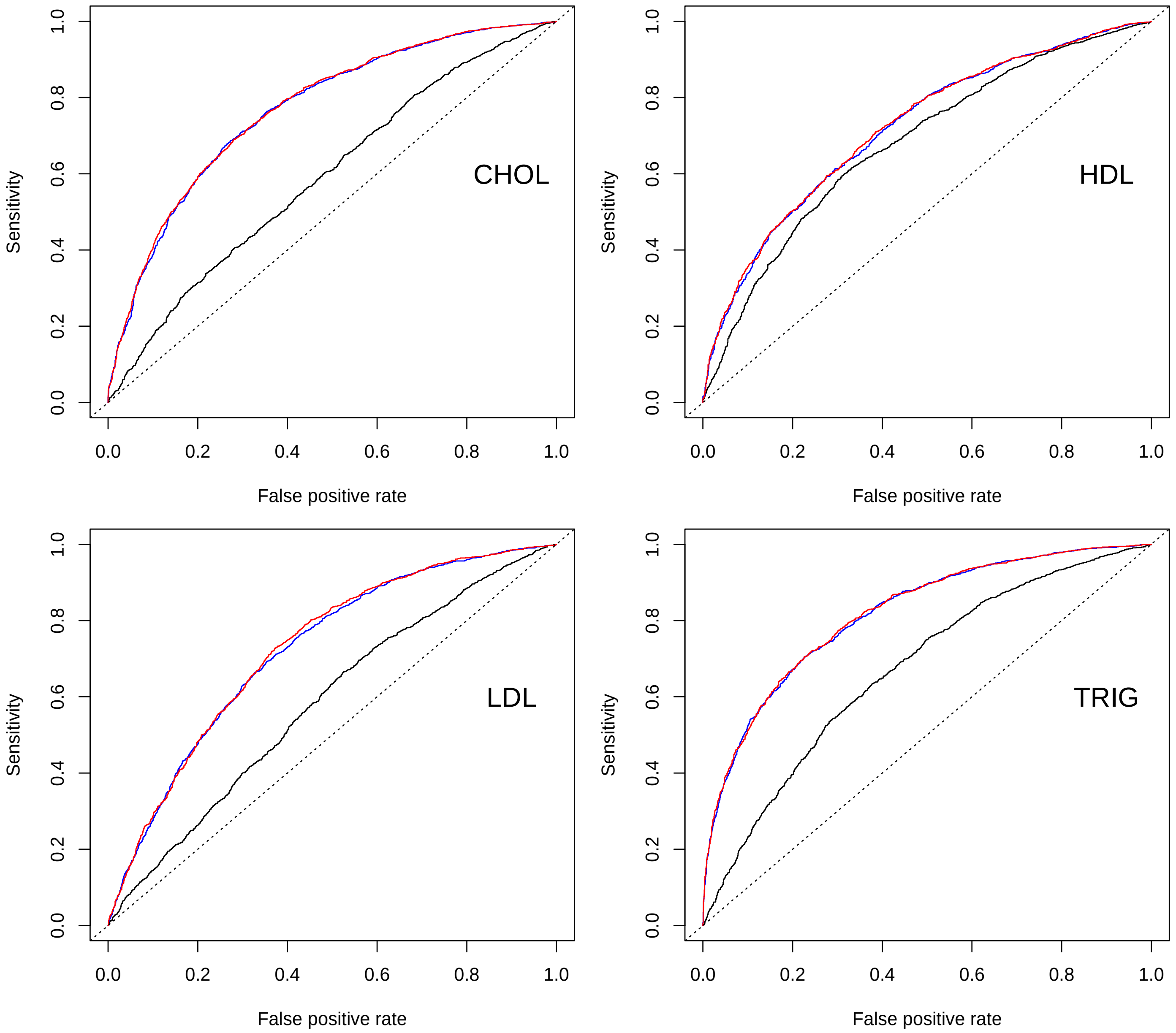

Figure S2. Receiver operating characteristic (ROC) curves for four phenotypes. The dotted line denotes the null curve. The black curve is for the model with only covariates. The blue curve is for the model with both covariates and phenotype-specific micronutrients. The red curve is for the model with environmental risk score (ERS), covariates and phenotype-specific micronutrients.
