# Supplementary material for: Environmental Risk Score as a New Tool to Examine Multi-Pollutants in Epidemiologic Research: An Example from the NHANES Study Using Serum Lipid Levels
Source: PLoS One. 2014 Jun 5;9(6):e98632. doi: 10.1371/journal.pone.0098632 (PMC4047033; doi:10.1371/journal.pone.0098632)
Supplement: Figure S3 — Odds ratios (95% confidence intervals) of having adverse levels of total cholesterol (CHOL: 200 mg/dL) and triglyceride (TRIG: 150 mg/dL) comparing the highest vs. the lowest quintiles of ERS and individual pollutants that compose the ERS. Models were adjusted for age, gender, race/ethnicity, education, BMI, and phenotype-specific micronutrients. (PDF) [file pone.0098632.s003.pdf]

Environmental Risk Score as a new tool to examine multi-pollutants in epidemiologic research: an example from the NHANES study using serum lipid levels

Sung Kyun Park, Yebin Tao, John D. Meeker, Siobán D. Harlow, Bhramar Mukherjee

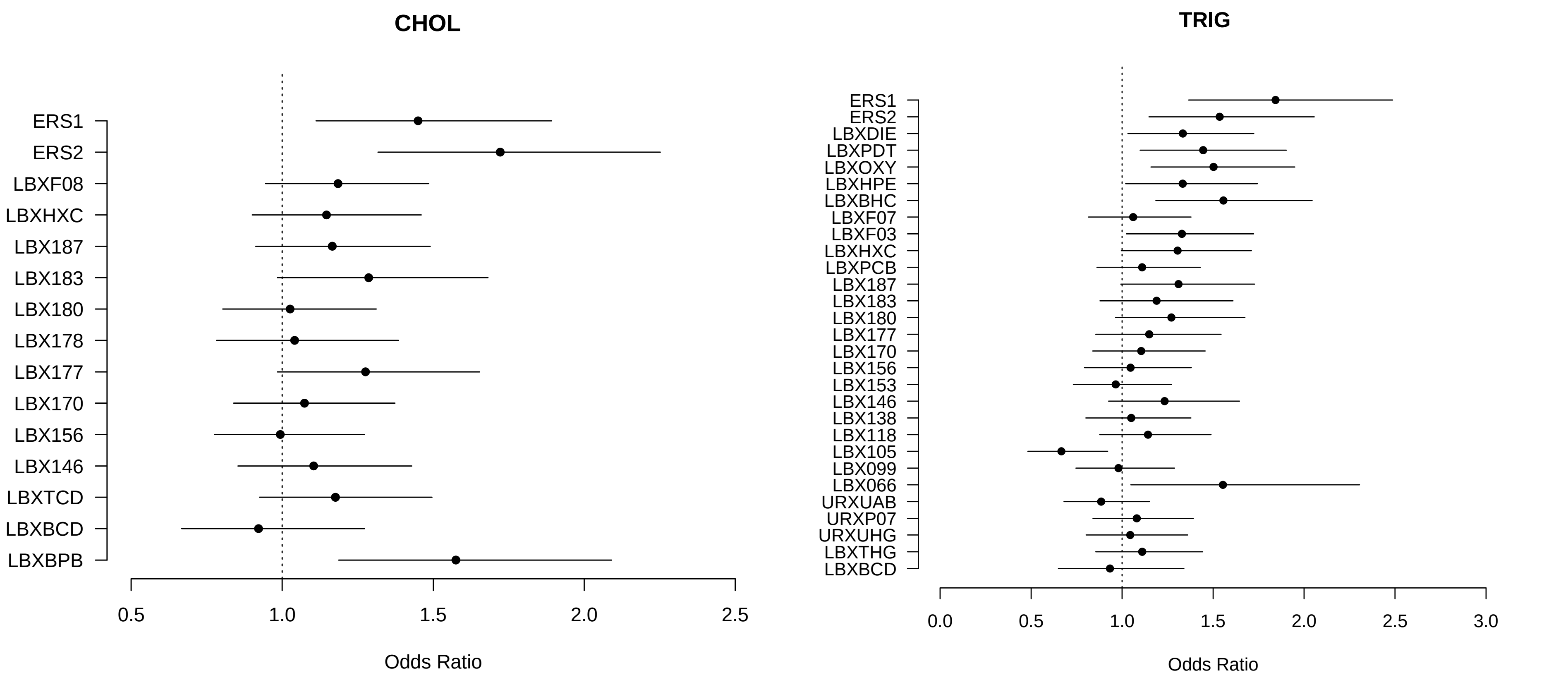

Figure S3. Odds ratios (95% confidence intervals) of having adverse levels of total cholesterol (CHOL: 200 mg/dL) and triglyceride (TRIG: 150 mg/dL) comparing the highest vs. the lowest quintiles of ERS and individual pollutants that compose the ERS. Models were adjusted for age, gender, race/ethnicity, education, BMI, and phenotype-specific micronutrients.

Note: LBXF08 (1,2,3,4,6,7,8-HpCDF), LBXHXC (3,3,4,4,5-HxCB), LBX187 (PCB 183), LBX180 (PCB 180), LBX178 (PCB 178), LBX177 (PCB 177), LBX170 (PCB 170), LBX156(PCB 156), LBX 146 (PCB 146), LBXTCD (2,3,7,8-TCDD), LBXBCD (Cadmium in blood), LBXBPB (Lead in blood), LBXDIE (Dieldrin), LBXPDT (*p*,*p*-DDT), LBXOXY (Oxychlordane), LBXHPE (Heptachlor Epoxide), LBXBHC (), LBXF07 (2,3,4,6,7,8-HxCDF), LBXF03 (2,3,4,7,8-PnCDF), LBXPCB (3,3,4,4,5,5-PnCB), LBX153(PCB 153), LBX138(PCB 138), LBX118(PCB 118), LBX105(PCB 105), LBX099(PCB 099), LBX066(PCB 066), URXUAB (Arsenobetaine in urine), URXP07 (2-phenanthrene), URXUHG (Mercury in urine), LBXTHG (Total mercury in blood).
