# Supplementary material for: Environmental Risk Score as a New Tool to Examine Multi-Pollutants in Epidemiologic Research: An Example from the NHANES Study Using Serum Lipid Levels
Source: PLoS One. 2014 Jun 5;9(6):e98632. doi: 10.1371/journal.pone.0098632 (PMC4047033; doi:10.1371/journal.pone.0098632)
Supplement: Table S1 — Environmental pollutants evaluated in the present study (n = 134). (PDF) [file pone.0098632.s004.pdf]

Environmental Risk Score as a new tool to examine multi-pollutants in epidemiologic research: an example from the NHANES study using serum lipid levels

*Sung Kyun Park, Yebin Tao, John D. Meeker, Siobán D. Harlow, Bhramar Mukherjee*

Table S1. Environmental pollutants evaluated in the present study (n=134).

| Class | N | Pollutant |
|---|---|---|
| Heavy metals | 22 | Lead (blood), lead (urine), cadmium (blood), cadmium (urine), total mercury (blood), inorganic mercury (blood), mercury (urine), total arsenic (urine), arsenous acid (urine), arsenic acid (urine), arsenic acid (urine), dimethylarsonic acid (urine), monomethylarsonic acid (urine), arsenobetaine (urine), arsenocholine (urine), barium (urine), cobalt (urine), cesium (urine), molybdenum (urine), antimony (urine),thallium (urine), tungsten (urine), uranium (urine) |
| Phthalates (urine) | 12 | mono-ethyl phthalate (MEP), mono-2-ethylhexyl phthalate (MEHP), mono-n-butyl phthalate (MBP), mono-(3-carboxylpropyl) phthalate (MCPP), mono-cyclohexyl phthalate (MCHP), mono-benzyl phthalate (MBzP), mono-n-octyl phthalate (MNOP), mono-isobutyl phthalate (MIBP), mono-isononyl phthalate (MINP), mono-2-ethyl-5-hydroxyhexyl phthalate (MEHHP), mono-2-ethyl-5-oxohexyl phthalate (MEOHP), mono-2-ethyl-5-carboxypentyl phthalate (MECPP) |
| Environmental phenols (urine) | 4 | Bisphenol-A (BPA), 4-tert-octylphenol, benzophenone-3, triclosan |
| Polycyclic aromatic hydrocarbons (PAHs) (urine) | 8 | 1-napthol, 2-napthol, 3-fluorene, 2-fluorene, 3-phenanthrene, 1-phenanthrene, 2-phenanthrene, 1-pyrene |
| Volatile organic compounds (VOCs) (blood) | 13 | Tetrachloroethene, Bromoform, Bromodichloromethane, Benzene, Chloroform, Dibromochloromethane, 1,4-Dichlorobenzene, Ethylbenzene, o-Xylene, Styrene, Trichloroethene, Toluene, m-/p-Xylene |
| Perfluorinated compounds (PFCs) (serum) | 12 | 2-(N-ethyl-PFOSA) acetate, 2-(N-methyl-PFOSA) acetate, Perfluorodecanoic acid, Perfluoroheptanoic acid, Perfluorohexane sulfonic acid, Perfluorononanoic acid, Perfluorooctanoic acid, Perfluorooctane sulfonic acid, Perfluorooctane sulfonamide, Perfluoroundecanoic acid, Perfluorobutane sulfonic acid, Perfluorododecanoic acid |
| Dioxins and furans (serum) | 12 | 1,2,3,7,8-PnCDD, 1,2,3,4,7,8-HxCDD, 1,2,3,6,7,8-HxCDD, 1,2,3,7,8,9-HxCDD, 1,2,3,4,6,7,8-HpCDD, 1,2,3,4,6,7,8,9-Octa-CDD, 2,3,7,8-TCDD, 2,3,4,7,8-PnCDF, 1,2,3,4,7,8-HxCDF, 1,2,3,6,7,8-HxCDF, 2,3,4,6,7,8-HxCDF, 1,2,3,4,6,7,8-HpCDF |
| Dioxin-like polychlorinated biphenyls (PCBs) (serum) | 10 | PCB 28, 66, 74, 105, 118, 156, 157, 167, 3,3,4,4,5,5-PnCB, 3,3,4,4,5-HxCB |
| Non-dioxin-like PCBs (serum) | 17 | PCB 52, 99, 101, 138-158, 146, 153, 170, 172, 177, 178, 180, 183, 187, 194, 196-203, 199, 206 |
| Organochlorine pesticides (serum) | 10 | Dichlorodiphenyldichloroethylene (*p,p'*-DDE), Dichlorodiphenyltrichloroethane (*p,p'*-DDT), *o,p'*-DDT, mirex, hexachlorobenzene (HCB), trans-nonachlor, beta-hexachlorocyclohexane (HCCH), oxychlordane, dieldrin, heptachlor epoxide |
| Organophosphate dialkyl metabolites (urine) | 6 | Dimethylphosphate, diethylphosphate, dimethyl-thiophosphate, diethyl-thiophosphate, dimethyl-dithiophosphate, diethyl-dithiophosphate |
| Herbicides (urine) | 3 | 2,4-D (2,4-dichlorophenoxyacetic acid), 2,4,5-T (2,4,5-trichlorophenoxyacetic acid), carbofuranphenol |
| Pesticides phenols (urine) | 5 | 2,5-dichlorophenol, 2,4,5-trichlorophenol, 2,4,6-trichlorophenol, 2,4-dichlorophenol, O-Phenyl phenol |
