# Supplementary material for: Environmental Risk Score as a New Tool to Examine Multi-Pollutants in Epidemiologic Research: An Example from the NHANES Study Using Serum Lipid Levels
Source: PLoS One. 2014 Jun 5;9(6):e98632. doi: 10.1371/journal.pone.0098632 (PMC4047033; doi:10.1371/journal.pone.0098632)
Supplement: Table S2 — Spearman correlation coefficients between four phenotypes. (PDF) [file pone.0098632.s005.pdf]

Environmental Risk Score as a new tool to examine multi-pollutants in epidemiologic research: an example from the NHANES study using serum lipid levels

Sung Kyun Park, Yebin Tao, John D. Meeker, Siobán D. Harlow, Bhramar Mukherjee

Table S2. Spearman correlation coefficients between four phenotypes.

	Total cholesterol	HDL	LDL	Triglyceride
Total cholesterol	1	0.155*	0.856*	0.372*
HDL		1	-0.027	-0.418*
LDL			1	0.148*
Triglyceride				1

*$p < 0.05$.
