# Supplementary material for: Environmental Risk Score as a New Tool to Examine Multi-Pollutants in Epidemiologic Research: An Example from the NHANES Study Using Serum Lipid Levels
Source: PLoS One. 2014 Jun 5;9(6):e98632. doi: 10.1371/journal.pone.0098632 (PMC4047033; doi:10.1371/journal.pone.0098632)
Supplement: Table S3 — Micronutrients selected for each phenotype using Bayesian model averaging (BMA). (PDF) [file pone.0098632.s006.pdf]

Environmental Risk Score as a new tool to examine multi-pollutants in epidemiologic research: an example from the NHANES study using serum lipid levels

Sung Kyun Park, Yebin Tao, John D. Meeker, Siobán D. Harlow, Bhramar Mukherjee

Table S3. Micronutrients selected for each phenotype using Bayesian model averaging (BMA).

Variable in NHANES	Name	Phenotype: Total cholesterol	HDL	LDL	Triglyceride
SFOL	Folate, serum (ng/mL)	x	x		x
SB12	Vitamin B12, serum (pg/mL)				x
RBCFOL	Folate, RBC (ng/mL RBC)	x		x	x
LBXMMA	Methylmalonic acid (μmol/L)	x		x	
LBXRPL	Retinyl palmitate (μg/dL)	x			x
LBXRST	Retinyl stearate (μg/dL)		x	x	x
LBXVIA	Retinol (μg/dL)	x	x	x	x
LBXBEC	Trans-β-carotene (μg/dL)	x	x	x	x
LBXCBC	Cis-β-carotene (μg/dL)	x	x		
LBXCRY	β-cryptoxanthin (μg/dL)	x			x
LBXLUZ	Combined lutein/zeaxanthin (μg/dL)	x	x	x	x
LBXLYC	Trans-lycopene (μg/dL)	x	x	x	
LBXATC	α-tocopherol (μg/dL)	x	x	x	x
LBXGTC	γ-tocopherol (μg/dL)	x	x	x	x
Total number included for adjustment		12	9	9	11

Note: A total of 31 micronutrient variables were considered. The variables not selected (n=17) are daidzein (URXDAZ), o-desmethylangolensin (URXDMA), equol (URXEQU), enterodiol (URXETD), enterolactone (URXETL), genistein (URXGNS), δ-tocopherol (LBXDTC), α-cryptoxanthin (LBXACY), α-carotene (LBXALC), total β-carotene (LBXBCC), cis-lycopene (LBXCLC), total lycopene (LBXLCC), cis-lutein/zeaxanthin (LBXCLZ), lutein (LBXLUT), zeaxanthin (LBXZEA), phytofluene (LBXPHF), and phytoene (LBXPHE).
