# Supplementary material for: Environmental Risk Score as a New Tool to Examine Multi-Pollutants in Epidemiologic Research: An Example from the NHANES Study Using Serum Lipid Levels
Source: PLoS One. 2014 Jun 5;9(6):e98632. doi: 10.1371/journal.pone.0098632 (PMC4047033; doi:10.1371/journal.pone.0098632)
Supplement: Table S4 — Distributions of Environmental Risk Scores (ERS) (n = 3847). (PDF) [file pone.0098632.s007.pdf]

Environmental Risk Score as a new tool to examine multi-pollutants in epidemiologic research: an example from the NHANES study using serum lipid levels

Sung Kyun Park, Yebin Tao, John D. Meeker, Siobán D. Harlow, Bhramar Mukherjee

Table S4. Distributions of Environmental Risk Scores (ERS) (n=3847).

	ERS1[a]		ERS2[b]	
	Mean (SD)	Range	Mean (SD)	Range
Total cholesterol	0.090 (0.043)	-0.068, 0.239	0.058 (0.019)	-0.009, 0.135
HDL	0.030 (0.057)	-0.226, 0.205	0.061 (0.022)	-0.013, 0.152
LDL	0.088 (0.029)	-0.059, 0.195	0.086 (0.027)	-0.054, 0.183
Triglyceride	-0.445 (0.228)	-1.278, 0.563	-0.009 (0.082)	-0.291, 0.339

HDL, high-density lipoprotein cholesterol; LDL, low-density lipoprotein cholesterol.
[a]ERS constructed with coefficient estimates from single-pollutant models as weights.
[b]ERS constructed with coefficient estimates from multi-pollutant models as weights.
