# Supplementary material for: Environmental Risk Score as a New Tool to Examine Multi-Pollutants in Epidemiologic Research: An Example from the NHANES Study Using Serum Lipid Levels
Source: PLoS One. 2014 Jun 5;9(6):e98632. doi: 10.1371/journal.pone.0098632 (PMC4047033; doi:10.1371/journal.pone.0098632)
Supplement: Table S5 — Risk prediction by continuous environmental risk score (ERS) using multi-phenotype approacha (n = 3847). (PDF) [file pone.0098632.s008.pdf]

Environmental Risk Score as a new tool to examine multi-pollutants in epidemiologic research: an example from the NHANES study using serum lipid levels

Sung Kyun Park, Yebin Tao, John D. Meeker, Siobán D. Harlow, Bhramar Mukherjee

Table S5. Risk prediction by continuous environmental risk score (ERS) using multi-phenotype approach[a] (n=3847).

Phenotype	Continuous Outcome						Dichotomized[b] Outcome		
	Model 1[c]		ERS1[d]		ERS2[e]		Model 1[c]	ERS1[d]	ERS2[e]
	R^2	PRESS[f]	R^2	PRESS[f]	R^2	PRESS[f]	AUC[g]	AUC[g]	AUC[g]
Total cholesterol	0.3277	122.49	0.3324	121.70	0.3344	121.34	0.7689 (0.7541, 0.7837)	0.7715 (0.7568, 0.7862)	0.7724 (0.7577, 0.7870)
HDL	0.2646	232.03	0.2696	230.57	0.2684	230.96	0.7200 (0.7031, 0.7368)	0.7232 (0.7065, 0.7399)	0.7232 (0.7065, 0.7400)
LDL	0.1384	538.78	0.1399	538.11	0.1400	538.06	0.7223 (0.7060, 0.7385)	0.7241 (0.7079, 0.7403)	0.7241 (0.7079, 0.7403)
Triglyceride	0.3717	967.68	0.3792	956.65	0.3789	957.15	0.8170 (0.8028, 0.8312)	0.8187 (0.8046, 0.8329)	0.8191 (0.8050, 0.8332)

[a]Pollutants selected by multi-phenotype regression (n = 45) to construct ERS which was computed in the validation data (n=3847), with adjustment for base covariates and union of selected micronutrients (n = 14).
[b]Continuous outcomes dichotomized to be high vs. low by thresholds: 200 mg/dL for total cholesterol, 40 mg/dL (male) or 50 mg/dL (female) for HDL, 130 mg/dL for LDL and 150 mg/dL for triglyceride.
[c]adjusted for base covariates and union of selected micronutrients for all outcomes.
[d]Model 1 plus ERS constructed with coefficient estimates from single-pollutant models as weights.
[e]Model 1 plus ERS constructed with coefficient estimates from multi-pollutant models as weights.
[f]Predicted residual sums of squares.
[g]Area under the receiver operating characteristic curve and 95% confidence interval(CI) computed with 2000 stratified bootstrap replicates.
