# Supplementary material for: Environmental Risk Score as a New Tool to Examine Multi-Pollutants in Epidemiologic Research: An Example from the NHANES Study Using Serum Lipid Levels
Source: PLoS One. 2014 Jun 5;9(6):e98632. doi: 10.1371/journal.pone.0098632 (PMC4047033; doi:10.1371/journal.pone.0098632)
Supplement: Table S6 — Regression outputs for each lipid outcome in relation to ERS1. (PDF) [file pone.0098632.s009.pdf]

Environmental Risk Score as a new tool to examine multi-pollutants in epidemiologic research: an example from the NHANES study using serum lipid levels

Sung Kyun Park, Yebin Tao, John D. Meeker, Siobán D. Harlow, Bhramar Mukherjee

Table S6. Regression outputs for each lipid outcome in relation to ERS1.

Total cholesterol

	Estimate	Std. Error	z value	Pr(>|z|)
(Intercept)	-22.8908	1.23094	-18.5960	< 2e-16
factor(ERS1.c)1	-0.1036	0.117226	-0.8840	0.376938
factor(ERS1.c)2	-0.0152	0.121588	-0.125	0.900721
factor(ERS1.c)3	0.1079	0.12672	0.851	0.394638
factor(ERS1.c)4	0.3719	0.1357	2.74	0.006138
sfol	-0.1646	0.095114	-1.73	0.083548
rbcfol	-0.2545	0.118771	-2.143	0.032112
LBXMMA	-0.1406	0.075971	-1.851	0.064137
LBXRPL	0.1114	0.048433	2.299	0.021494
LBXVIA	0.7174	0.138545	5.178	2.24E-07
LBXBEC	-0.0239	0.103841	-0.231	0.817681
LBXCBC	0.0176	0.121434	0.145	0.884473
LBXCRY	0.2250	0.074419	3.024	0.002497
LBXGTC	0.8542	0.070168	12.174	< 2e-16
LBXLUZ	0.5120	0.094595	5.413	6.21E-08
LBXLYC	0.4502	0.070091	6.423	1.34E-10
lbxatc	1.8105	0.120479	15.027	< 2e-16
age	0.0064	0.002622	2.431	0.015044
gender	0.3863	0.078959	4.892	9.98E-07
factor(raceeth)2	-0.1684	0.193873	-0.869	0.384954
factor(raceeth)3	0.1129	0.114634	0.985	0.324549
factor(raceeth)4	-0.2397	0.12441	-1.927	0.054034
factor(raceeth)5	0.0556	0.211333	0.263	0.792477
factor(educat)2	-0.1396	0.105265	-1.327	0.184638
factor(educat)3	-0.1830	0.096644	-1.894	0.058284
factor(cycle)2	-0.1939	0.107031	-1.812	0.070059
factor(cycle)3	-0.2481	0.112527	-2.205	0.027488
factor(cycle)4	-0.4086	0.120488	-3.391	0.000696
bmi	0.0129	0.00605	2.134	0.032848

HDL

	Estimate	Std. Error	z value	Pr(>|z|)
(Intercept)	-1.8167	0.94587	-1.921	0.054768
factor(ERS1.c)1	0.0659	0.114065	0.578	0.563566
factor(ERS1.c)2	-0.1595	0.116614	-1.367	0.171486
factor(ERS1.c)3	-0.1323	0.118026	-1.121	0.262269
factor(ERS1.c)4	-0.3164	0.123549	-2.561	0.010448
sfol	-0.2626	0.082123	-3.198	0.001386
LBXRST	0.4252	0.066135	6.43	1.28E-10

LBXVIA	0.0100	0.133673	0.075	0.940127
LBXBEC	0.3390	0.095798	3.538	0.000403
LBXCBC	-0.4568	0.119803	-3.813	0.000137
LBXGTC	0.2134	0.066923	3.189	0.001427
LBXLUZ	-0.7847	0.089902	-8.728	< 2e-16
LBXLYC	-0.2335	0.067412	-3.464	0.000533
lbxatc	0.2503	0.107001	2.339	0.019315
age	-0.0073	0.002344	-3.096	0.001959
gender	0.2111	0.079602	2.652	0.008009
factor(raceeth)2	0.3350	0.181307	1.848	0.064669
factor(raceeth)3	-0.2092	0.104754	-1.997	0.045868
factor(raceeth)4	-0.6673	0.12195	-5.472	4.45E-08
factor(raceeth)5	-0.0903	0.210313	-0.429	0.667615
factor(educat)2	-0.1645	0.104175	-1.579	0.114257
factor(educat)3	-0.4127	0.096838	-4.261	2.03E-05
factor(cycle)2	-0.2725	0.104385	-2.611	0.009041
factor(cycle)3	-0.2783	0.112895	-2.465	0.013686
factor(cycle)4	-0.6027	0.121072	-4.978	6.43E-07
bmi	0.0653	0.006233	10.471	< 2e-16

LDL

	Estimate	Std. Error	z value	Pr(>|z|)
(Intercept)	-16.8923	1.141381	-14.8	< 2e-16
factor(ERS1.c)1	0.1423	0.119816	1.188	0.234993
factor(ERS1.c)2	0.2941	0.1223	2.405	0.016167
factor(ERS1.c)3	0.3816	0.127062	3.003	0.00267
factor(ERS1.c)4	0.6010	0.137007	4.387	1.15E-05
rbcfol	-0.3346	0.101103	-3.31	0.000934
LBXMMA	-0.1826	0.075774	-2.41	0.01597
LBXRST	-0.3316	0.064328	-5.155	2.53E-07
LBXVIA	0.3701	0.1371	2.7	0.006939
LBXBEC	0.1752	0.050981	3.436	0.000591
LBXGTC	0.6364	0.066129	9.624	< 2e-16
LBXLUZ	0.4230	0.086103	4.912	9.00E-07
LBXLYC	0.4859	0.070176	6.924	4.40E-12
lbxatc	1.2785	0.113995	11.216	< 2e-16
age	0.0042	0.002508	1.659	0.097068
gender	-0.0283	0.078473	-0.36	0.718653
factor(raceeth)2	0.0933	0.190293	0.49	0.623942
factor(raceeth)3	0.2909	0.105861	2.748	0.006003
factor(raceeth)4	-0.0371	0.118581	-0.312	0.754683
factor(raceeth)5	-0.0238	0.207474	-0.115	0.908733
factor(educat)2	-0.0516	0.103349	-0.499	0.617639
factor(educat)3	-0.1996	0.095965	-2.08	0.037563
factor(cycle)2	-0.2535	0.102219	-2.48	0.01315

factor(cycle)3	-0.6121	0.111406	-5.494	3.92E-08
factor(cycle)4	-0.6281	0.120591	-5.208	1.90E-07
bmi	0.0150	0.005978	2.511	0.012047

Triglyceride

	Estimate	Std. Error	z value	Pr(>|z|)
(Intercept)	-20.7623	1.391222	-14.924	< 2e-16
factor(ERS1.c)1	0.2078	0.13939	1.491	0.135954
factor(ERS1.c)2	0.1799	0.142209	1.265	0.205883
factor(ERS1.c)3	0.3354	0.145443	2.306	0.021121
factor(ERS1.c)4	0.6114	0.152866	3.999	6.35E-05
sfol	-0.4172	0.106799	-3.907	9.36E-05
sb12	-0.4669	0.093393	-4.999	5.76E-07
rbcfol	0.5345	0.132454	4.035	5.45E-05
LBXRPL	0.2634	0.064914	4.058	4.95E-05
LBXRST	0.8999	0.091732	9.81	< 2e-16
LBXVIA	1.1813	0.156471	7.549	4.37E-14
LBXBEC	-0.3468	0.065643	-5.284	1.27E-07
LBXCRY	0.0584	0.082382	0.709	0.478405
LBXGTC	0.8281	0.076675	10.801	< 2e-16
LBXLUZ	-0.2239	0.10552	-2.122	0.033811
lbxatc	1.8307	0.132078	13.861	< 2e-16
age	-0.0096	0.002758	-3.475	0.000512
gender	0.0300	0.086996	0.345	0.730106
factor(raceeth)2	-0.2787	0.207651	-1.342	0.179486
factor(raceeth)3	-0.6261	0.124681	-5.022	5.12E-07
factor(raceeth)4	-1.2028	0.144784	-8.308	< 2e-16
factor(raceeth)5	0.1397	0.224593	0.622	0.533933
factor(educat)2	-0.3797	0.116447	-3.261	0.001111
factor(educat)3	-0.4305	0.107113	-4.019	5.84E-05
factor(cycle)2	-0.1089	0.118175	-0.921	0.356876
factor(cycle)3	0.2682	0.128869	2.082	0.037387
factor(cycle)4	0.0336	0.130944	0.257	0.79755
bmi	0.0589	0.006931	8.492	< 2e-16
