# Supplementary material for: Environmental Risk Score as a New Tool to Examine Multi-Pollutants in Epidemiologic Research: An Example from the NHANES Study Using Serum Lipid Levels
Source: PLoS One. 2014 Jun 5;9(6):e98632. doi: 10.1371/journal.pone.0098632 (PMC4047033; doi:10.1371/journal.pone.0098632)
Supplement: File S1 — Diagnostic Analysis for the Imputation. (PDF) [file pone.0098632.s010.pdf]

Environmental Risk Score as a new tool to examine multi-pollutants in epidemiologic research: an example from the NHANES study using serum lipid levels

Sung Kyun Park, Yebin Tao, John D. Meeker, Siobán D. Harlow, Bhramar Mukherjee

File S1. Diagnostic Analysis for the Imputation.

1. Summary statistics of imputed data and cross-correlation across pollutants. Following table is some sample output.

Summary statistics:

Before imputation				
Blood lead	Blood cadmium	Urinary cadmium	Serum folate	Serum Vit B12
Min. :-2.30568	Min. :-3.3589	Min. :-4.3710	Min. :0.6931	Min. : 3.526
1st Qu.: 0.09531	1st Qu.:-1.6094	1st Qu.:-1.8816	1st Qu.:2.1163	1st Qu.: 5.869
Median : 0.47000	Median :-0.9163	Median :-1.2379	Median :2.4596	Median : 6.142
Mean : 0.51160	Mean :-0.9346	Mean :-1.2298	Mean :2.4796	Mean : 6.155
3rd Qu.: 0.95551	3rd Qu.:-0.3567	3rd Qu.:-0.5621	3rd Qu.:2.8154	3rd Qu.: 6.418
Max. : 3.53514	Max. : 2.0015	Max. : 2.9788	Max. :6.5352	Max. :10.442
After imputation				
Blood lead	Blood cadmium	Urinary cadmium	Serum folate	Serum Vit B12
Min. :-2.30568	Min. :-3.3589	Min. :-8.3994	Min. :-0.3567	Min. : 3.526
1st Qu.: 0.04879	1st Qu.:-1.5099	1st Qu.:-1.8429	1st Qu.: 2.1633	1st Qu.: 5.855
Median : 0.48243	Median :-0.9163	Median :-1.2081	Median : 2.4932	Median : 6.140
Mean : 0.50319	Mean :-0.8796	Mean :-1.2155	Mean : 2.5137	Mean : 6.151
3rd Qu.: 0.95551	3rd Qu.:-0.5108	3rd Qu.:-0.5538	3rd Qu.: 2.8507	3rd Qu.: 6.425
Max. : 3.97029	Max. : 2.3795	Max. : 3.6049	Max. : 6.5352	Max. :11.907

Correlation across variables:

Before					
	Blood lead	Blood cadmium	Urinary cadmium	Serum folate	Serum Vit B12
Blood Pb	1	0.340404771	0.298852239	-0.07801443	0.039259208
Blood Cd	0.34040477	1	0.457522373	-0.07122365	0.007823718
Urine Cd	0.29885224	0.457522373	1	-0.03239455	-0.004978353
S folate	-0.07801443	-0.071223653	-0.03239455	1	0.223723737
S Vit B12	0.03925921	0.007823718	-0.004978353	0.22372374	1
After					
	Blood lead	Blood cadmium	Urinary cadmium	Serum folate	Serum Vit B12
Blood Pb	1	0.342744657	0.31465779	-0.08846296	0.031779435
Blood Cd	0.34274466	1	0.4451359	-0.06502275	-0.001286846
Urine Cd	0.31465779	0.445135899	1	-0.03655016	-0.011661582
S folate	-0.08846296	-0.06502275	-0.03655016	1	0.214452768
S Vit B12	0.03177944	-0.001286846	-0.01166158	0.21445277	1

2. Comparison of the distribution of pollutants before vs. after imputation. For example we present results for two pollutants with different degree of missingness: LBXTHG (total mercury) (43% missing) and LBXTCD (2,3,7,8-TCDD) (82% missing) below with the black curve denoting density before imputation and the red curve showing density after imputation. As one can note the quality of imputation worsens as the % missing data increases, as expected.

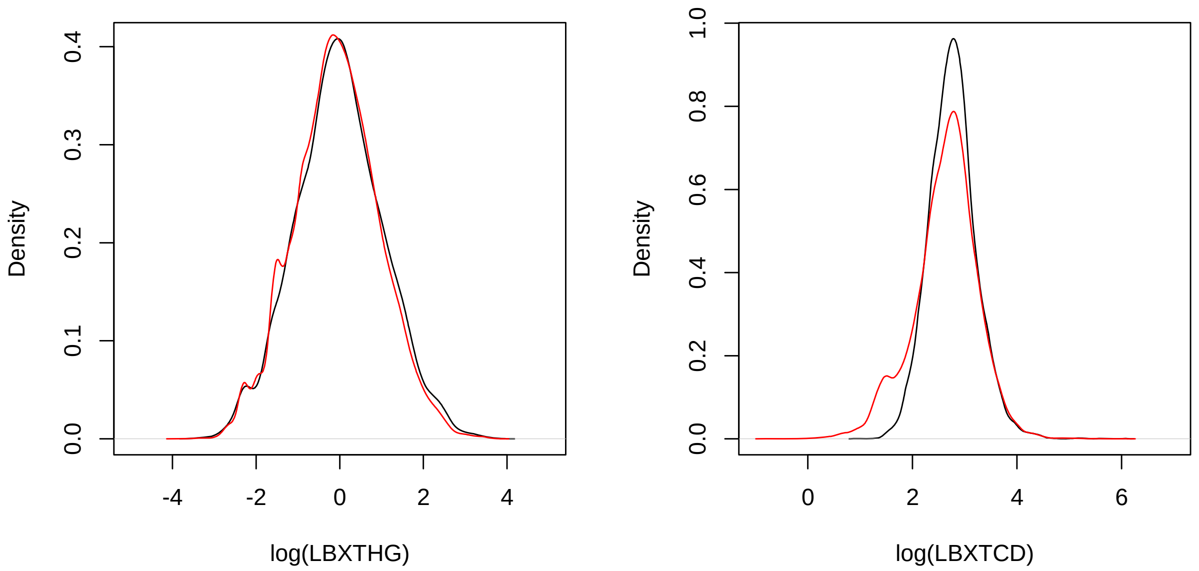

Figure A. Distribution of LBXTHG (total mercury) (left) and LBXTCD (2,3,7,8-TCDD) (right) before (black curve) vs. after (red curve) imputation.

3. Assessment of how observed data compare when they are hidden artificially and imputed. To this end we removed 100 observations from two examples, LBXTHG (43% missingness) and LBXTCD (82% missingness), and compared observed data with imputed values. The correlation between observed and imputed values was 0.82 for LBXTHG and 0.58 for LBXTCD. There is some bias in the case with larger missingness, as shown by the departure from the line of y = x in the figure below), However, since we are carrying out a linear association analysis, preserving strong correlation of imputed data with observed data is more important in our context.

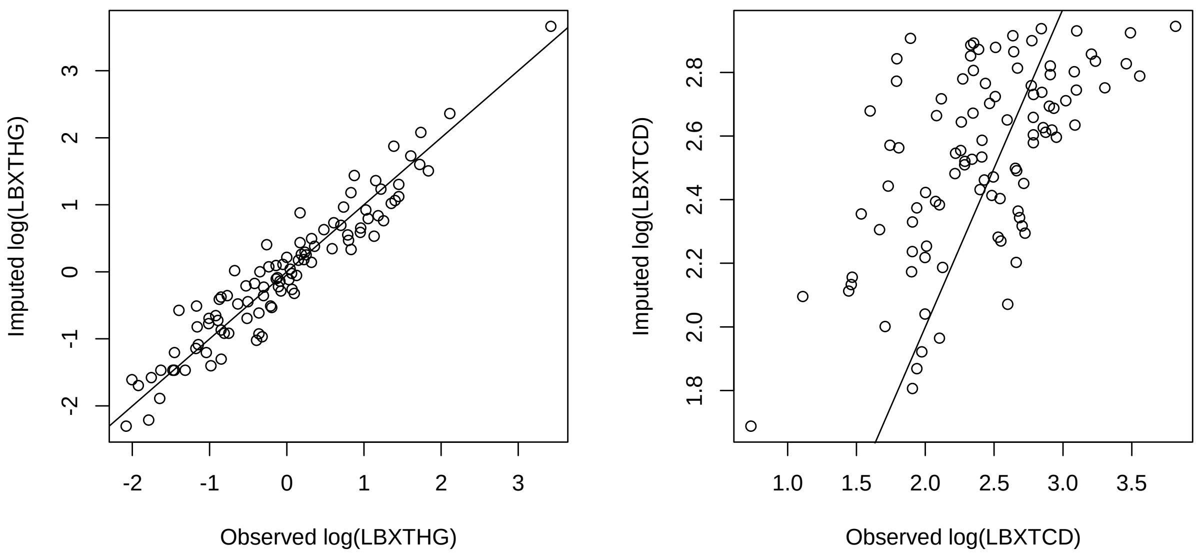

Figure B. A comparison of imputed values and the pre-removed observations for LBXTHG (total mercury) and LBXTCD (2,3,7,8-TCDD); the line is y = x.
